# Room-temperature formation of CdS magic-size clusters in aqueous solutions assisted by primary amines

**DOI:** 10.1038/s41467-020-18014-6

**Published:** 2020-08-21

**Authors:** Wushuang Wan, Meng Zhang, Min Zhao, Nelson Rowell, Chunchun Zhang, Shanling Wang, Theo Kreouzis, Hongsong Fan, Wen Huang, Kui Yu

**Affiliations:** 1 School of Chemical Engineering, Sichuan University, 610065 Chengdu, PR China; 2 Institute of Atomic and Molecular Physics, Sichuan University, 610065 Chengdu, PR China; 3 Metrology Research Centre, National Research Council of Canada, Ottawa, ON K1A 0R6 Canada; 4 Analytical & Testing Center, Sichuan University, 610065 Chengdu, PR China; 5 School of Physics and Astronomy, Queen Mary University of London, London, E1 4NS UK; 6 Engineering Research Center in Biomaterials, Sichuan University, 610065 Chengdu, PR China; 7 Laboratory of Ethnopharmacology, West China School of Medicine, West China Hospital, Sichuan University, 610065 Chengdu, PR China; 8 State Key Laboratory of Polymer Materials Engineering, Sichuan University, 610065 Chengdu, PR China

**Keywords:** Inorganic chemistry, Physical chemistry, Nanoparticles

## Abstract

Aqueous-phase approaches to semiconductor CdS magic-size clusters (MSCs) and the formation pathway have remained relatively unexplored. Here, we report the demonstration of an aqueous-phase, room-temperature approach to CdS MSCs, together with an exploration of their evolution pathway. The resulting CdS MSCs display a sharp optical absorption peak at about 360 nm and are labeled MSC-360. With CdCl_2_ and thiourea as the respective Cd and S sources, and 3-mercarpotopropionic acid as the ligand, CdS MSC-360 develops in a mixture of a primary amine and water. We argue that the primary amine facilitates room-temperature decomposition of thiourea when CdCl_2_ is present, and the formation pathway of MSCs is similar to that in organic-phase approaches. Our findings show there is a viable avenue to room-temperature aqueous-phase formation of CdS MSCs. Providing explanations of the procedure developed including the formation of large aggregates, the present study represents an important advance towards a mechanistic understanding of nanocrystal synthesis.

## Abstract (web summary)

CdS magic-size clusters have, so far, been prepared only in organic solvents. Here, the authors report an aqueous-phase synthesis for CdS magic-size clusters at room temperature and reveal insights into the formation mechanism, including the key role of primary amines.

## Introduction

Crystallization from liquid phases is acknowledged to be central to materials science and vital in applications such as pharmaceutical and chemical manufacturing. In this regard, two models have been developed for nucleation, which are one-step or multistep based^1–28^. The one-step model, described by classical nucleation theory (CNT), involves the concept of supersaturation or supercooling^1–3^. The multistep model, designated by nonclassical nucleation theory, accounts for the presence of intermediates in the pre-nucleation stage^4–28^. Such intermediates have been illustrated in several materials systems including calcium-based inorganics^4–8^, organics^9–11^, polymers^12–14^, metals^15–17^, and semiconductor quantum dots (QDs)^18–28^.

For organic-phase approaches to semiconductor metal (M) chalcogenide (E) QDs, a two-pathway model has been proposed for the pre-nucleation stage^18–28^. One pathway involves the formation of monomers and fragments, which result in QDs as per the LaMer model of the CNT. The other pathway deals with the self-assembly of M and E precursors which occurs first followed by the formation of particular precursor compounds (PCs) for magic-size clusters (MSCs). The self-assembly pathway proposed for the MSC formation is consistent with the high-concentration approach to CdS MSCs^29^. These two individual pathways are interconnected; when QDs grow in size, fragmentation of PCs into monomers and fragments happens. Usually, MSCs exhibit relatively sharp optical absorption peaking at a persistent position compared to their corresponding QDs, while the PCs display no characteristic optical absorption. The PC is conceptually the same as the oligomer (CaCO_3_)_*n*_ (*n* = 3–11) and Posner molecule (Ca_3_(PO_4_)_2_)_3_ reported for the pre-nucleation stage of calcium-based inorganics^4,7,28^. The PC to MSC transformation occurs via intramolecular reorganization, which follows first-order reaction kinetics^19,23,26^. A PC and its corresponding MSC appear to be one polymorphous pair, with similar semiconductor cores but different organic ligands. The PC to MSC transformation has been demonstrated only in nonaqueous-phase approaches for semiconductors, including CdS^18–20^, CdSe^21,25^, CdTe^22–25^, CdTeSe^25^, ZnS^26^, and ZnSe^27^.

A few examples of aqueous-phase approaches for the synthesis of colloidal semiconductor QDs and MSCs have been reported, while these studies have been largely empirical^30–41^. The complexity of aqueous environments including ionic interactions has been claimed to inhibit understanding the formation pathways of QDs and MSCs^30,37^. Supplementary Tables 1 and 2 summarize the published experimental results regarding aqueous-phase syntheses of CdE MSCs and QDs, respectively. Until the present work only CdSe MSCs, which exhibit sharp optical absorption peaking at about 420 nm with full width at half maximum of 20 nm, have been synthesized^38–41^. The approach to the evolution of the CdSe MSCs requires long reaction times (such as 7 days), and the Se source Na_2_SeSO_3_ has to be freshly prepared due to its limited stability. The aqueous-phase synthesis of CdS MSCs has not been demonstrated, and whether the formation pathway of aqueous-phase MSCs is similar to that of nonaqueous phase ones is not known^18–28^.

Here, we present the aqueous-phase approach to CdS MSCs and describe our understanding of the procedure developed and of the formation pathway. With CdCl_2_ and thiourea (TU, S = C(NH_2_)_2_) as the respective Cd and S sources with the feed molar ratio of 2 to 1, and 3-mercarpotopropionic acid (MPA, HS–CH_2_CH_2_–COOH) as the ligand, CdS MSCs in a single-ensemble form evolve in a mixture of a primary amine and water. The resulting MSCs exhibit sharp optical absorption peaking at about 360 nm, and are denoted as MSC-360 (in reference to their absorption peak position in wavelength). Importantly, the presence of a primary amine, such as butylamine (BTA, CH_3_–(CH_2_)_3_–NH_2_), is a prerequisite for the evolution of CdS MSC-360 at room temperature. BTA assists room-temperature decomposition of TU upon the presence of CdCl_2_. And the quantity of CdCl_2_ has a very significant influence; when the Cd concentration is above 4.0 mM, the formation of MSC-360 is suppressed due to the presence of large aggregates (above 100 nm). The higher the concentration is, the larger the aggregates are. We demonstrate that for an aqueous solution of CdCl_2_ and MPA in a basic environment (pH = ~12), aggregation occurs when the Cd concentration is above 4.0 mM. The resulting aggregates have relatively hydrophobic cores, as indicated by fluorescence spectroscopy of pyrene. Nuclear magnetic resonance (NMR) spectroscopy provides further evidence for the formation and aggregation of Cd−MPA complexes. Moreover, we show that purified CdS MSC-360 disappears gradually in deionized water, but reappears after a primary amine is added. This behavior indicates that a reversible PC ⇔ MSC transformation takes place, which is similar to that in the nonaqueous approaches^18–28^. The present findings introduce a room-temperature aqueous-phase approach to the production of single-ensemble CdS MSC-360 without the co-production of QDs, clarifying that the PC to MSC transformation is applicable for the aqueous-phase formation of semiconductor MSCs. The present study contributes to the advances of cluster science and the science of crystallization.

## Results

### Aqueous-phase evolution of CdS MSC-360

**Fig. 1:**
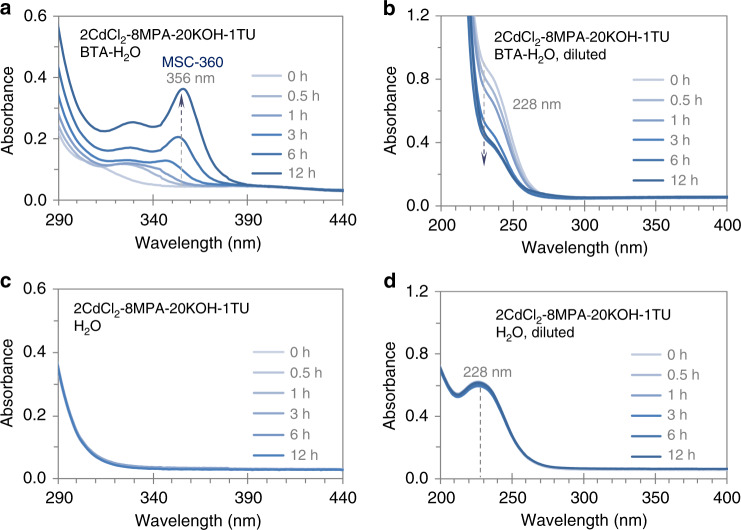
Absorption spectra of one mixture of CdCl_2_, MPA, KOH, and TU in two solvents. They are a mixture of BTA and H_2_O with equal volumes (**a**, **b**), and H_2_O (**c**, **d**). The spectra are collected at the various periods indicated (**a**, **c**); right after each measurement, an aliquot (50 μL) is diluted in 3.0 mL of water (**b**, **d**). The spectra are offset to have similar absorbance at 440 (**a**, **c**) or 400 nm (**b**, **d**). The dashed arrows (**a**, **b**) and line (**d**) signify the positions indicated. Evidently, the presence of BTA is a prerequisite for the room-temperature evolution of CdS MSC-360.

Figure 1 presents the evolution of optical absorption spectra collected from one mixture in two solvents. The mixture contains CdCl_2_ (2.0 mM), MPA (8.0 mM), KOH (20.0 mM), and TU (1.0 mM). One solvent is a mixture of BTA (1.5 mL) and water (H_2_O, 1.5 mL) (Fig. 1a, b). The other solvent is H_2_O (3.0 mL) (Fig. 1c, d). For the BTA-water solution, the mixture is first placed in 1.5 mL of water followed by the addition of 1.5 mL of BTA. See the “Methods” section for details regarding the preparation of the two solutions. The absorption spectra are collected after elapsed times of 0, 0.5, 1, 3, 6, and 12 h (Fig. 1a, c); at each time point, an aliquot (50 μL) is extracted and diluted in deionized water (3.0 mL) and absorption measurements are performed (Fig. 1b, d).

In the mixture of BTA and water (Fig. 1a), an absorption feature gradually develops to peak at ~356 nm, which indicates the presence of CdS MSC-360. At the beginning (0 h), the spectrum is quite featureless. At 3 h, a peak at 347 nm can be identified clearly. Afterwards, this peak increases in intensity considerately and red shifts slightly to 356 nm at the 12 h point. Simultaneously, the intensity of the absorption around 228 nm decreases (Fig. 1b). Supplementary Fig. 1 suggests such short wavelength absorption is due to CdCl_2_, MPA, and TU together. Accordingly, the overall conversion yield is estimated to be about 60% after a period of 6 h (as illustrated by Supplementary Fig. 2). In water (Fig. 1c), the absorption spectra are featureless, which suggests that little reaction takes place. Also, the absorption at 228 nm remains constant (Fig. 1d), in agreement with that there is no consumption of the Cd and S sources.

Therefore, the evolution of CdS MSC-360 at room temperature occurs only when the mixture of CdCl_2_, MPA, KOH, and TU is in the BTA and water environment (Fig. 1a), in which case we observe the consumption of the starting materials (Fig. 1b). When this mixture is placed in water without BTA, the starting materials are not consumed and the evolution of CdS MSC-360 does not take place (Fig. 1c, d). Figure 1a, c focuses on the possible development of MSCs, while Fig. 1b, d illustrates the likely consumption of the reactants. The synthesis conditions have been carefully optimized, such as the Cd to S feed molar ratio (Supplementary Fig. 3 with a constant Cd concentration of 2.0 mM). Clearly, the characteristic optical absorption peak of MSC-360 broadens gradually upon increasing the TU concentration. In this regard we use a 2CdCl_2_ to 1TU feed molar ratio to synthesize CdS MSC-360.

Transmission electron microscopy (TEM), powder X-ray diffraction (XRD), and thermogravimetric analysis (TGA) have been employed (Supplementary Fig. 4). Although the conventional characterization tools have some shortcomings with regard to providing precise structural and compositional information of colloidal semiconductor small-size QDs and MSCs^20,25–28^, Supplementary Fig. 4 suggests that the aqueous-phase CdS MSCs are spherical with a diameter smaller than 3 nm, and have a similar structure as that of organic-phase CdS MSC-361^42^, with the ligand to inorganic core weight ratio of 20 to 80 (Supplementary Note 1). The Fourier transform infrared (FT-IR) spectrum of the purified CdS MSC-360 sample (Supplementary Fig. 5) indicates that the purified MSCs are passivated only by MPA molecules which act as surface ligands. Therefore, there is no evidence that these MSCs have any of the primary amine, BTA, associated with them.

### Effect of amounts and nature of primary amines

**Fig. 2:**
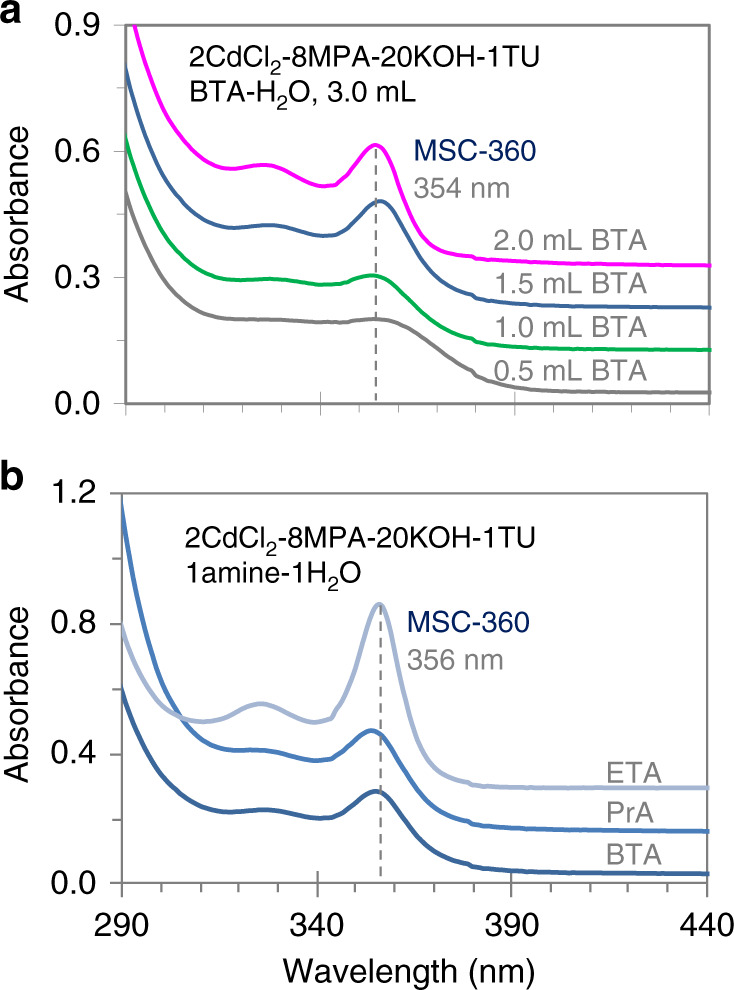
Absorption spectra (offset) of the same mixture in different solvents. **a** The four solvents are 3.0 mL of BTA and water mixtures, with 0.5 (gray trace), 1.0 (green trace), 1.5 (blue trace), and 2.0 (magenta trace) mL BTA. **b** The three solvents are the mixtures of 1.5 mL primary amines (BTA (blue trace), PrA (lighter blue trace), and ETA (lightest blue trace)) and 1.5 mL water. All the spectra are collected from the solutions kept in cuvettes at room temperature for 24 h. The dashed lines signify the positions indicated. Evidently, increasing the amount of BTA, or using a primary amine with a shorter alkyl chain, facilitates the production of MSC-360.

For the formation of MSC-360 in the mixture of CdCl_2_, MPA, KOH, and TU, Fig. 1 suggests that BTA plays an important role. In Fig. 2, we present the effects of the quantity of BTA (Fig. 2a) and of different primary amines (Fig. 2b). The absorption spectra are collected from the same mixture but in four solvents containing four different amounts of BTA (Fig. 2a) and in three solvents with three different primary amines (Fig. 2b). The mixture consists of CdCl_2_ (2.0 mM), MPA (8.0 mM), KOH (20.0 mM), and TU (1.0 mM); after the mixture is placed into water, a primary amine is added to result in a final volume of 3.0 mL. In Fig. 2a, the added BTA is 0.5 (gray trace), 1.0 (green trace), 1.5 (blue trace), and 2.0 mL (magenta trace). In Fig. 2b, 1.5 mL of a primary amine of BTA (blue trace), propylamine (PrA, CH_3_–(CH_2_)_2_–NH_2_, lighter blue trace), and ethylamine (ETA, CH_3_–CH_2_–NH_2_, lightest blue trace) is added. Supplementary Fig. 6 presents their chemical structures and 3D models. All the spectra are collected 24 h after the preparation of the solutions at room temperature. The temporal evolution of the absorption properties of these solutions are shown in Supplementary Figs. 7 (BTA), 8 (PrA), and 9 (ETA).

In Fig. 2a, the absorption spectrum of the solution having 2.0 mL BTA displays a sharp optical absorption peaking at about 354 nm, while that of the solution with 0.5 mL BTA exhibits a relatively broad absorption. Supplementary Fig. 7 demonstrates that CdS MSCs with a sharper absorption are produced at 24 h in a solvent with more BTA. This seems to be the case with PrA as well (Supplementary Fig. 8). When 1.5 mL of ETA is added instead of BTA or PrA, more CdS MSC-360 is present at 24 h (Fig. 2b). Solutions with amine volumes up to 1.5 mL are relatively transparent at all times, and MSC-360 appears gradually (Supplementary Figs. 7–9). Solutions with 2.0 mL of BTA or ETA appear milky at the start and become clear after around 12 or 24 h, respectively, and the evolution of MSC-360 is observed (Supplementary Figs. 7d and 9d). Because of the precipitation (with 2.0 mL of amine) and the evaporation of PrA (boiling point 48 °C) and ETA (boiling point 17 °C), the mixture of BTA (1.5 mL), and water (1.5 mL) is used in this study to synthesize CdS MSC-360.

It is noteworthy that BTA has been claimed to assist the decomposition of a TU derivative in N,N-dimethylformamide (DMF) during the nucleation and growth of PbS nanocrystals^43^. In the absence of CdCl_2_, Supplementary Fig. 10 suggests that TU remains stable at room temperature in a mixture of BTA (1.5 mL) and water (1.5 mL). In this regard the room-temperature decomposition of TU requires both BTA and the Cd precursor. Moreover, only primary amines are found to facilitate the room-temperature evolution of CdS MSC-360. For example, when a secondary amine diethylamine is used (Supplementary Fig. 11), no CdS MSC-360 is observed.

### CdCl_2_ concentration effect upon aggregation

**Fig. 3:**
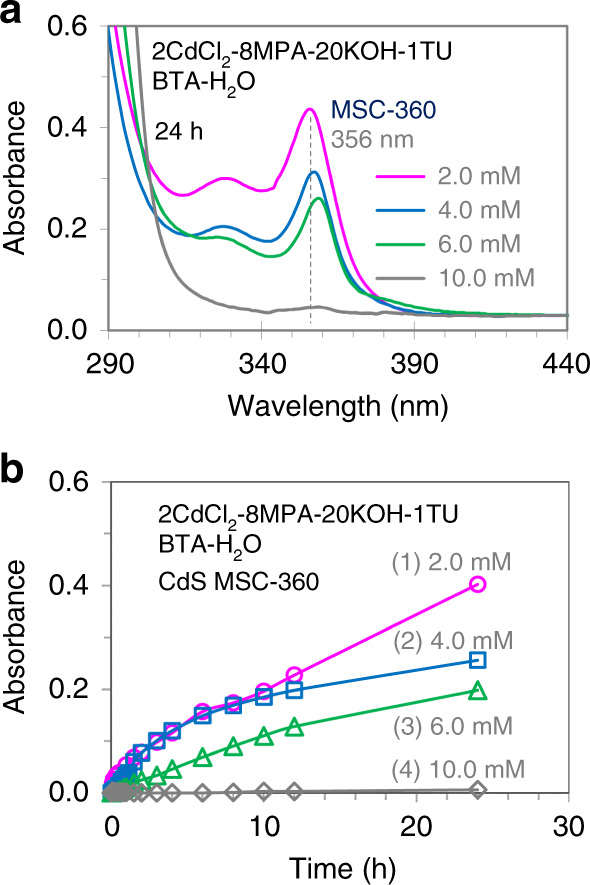
Absorption properties of four solutions with four CdCl_2_ concentrations. For the four solutions, the absorption spectra after 24 h of their preparation are shown in **a** and time-dependent absorbance of MSC-360 is summarized in **b**. In the solvent of BTA (1.5 mL) and water (1.5 mL), the four mixtures have the feed molar ratio of 2CdCl_2_-8MPA-20KOH-1TU with the CdCl_2_ concentrations of 2.0 (magenta trace for **a** and circular symbols for **b**), 4.0 (blue trace for **a** and square symbols for **b**), 6.0 (green trace for **a** and triangular symbols for **b**), and 10.0 mM (gray trace for **a** and diamond symbols for **b**). The spectra (**a**) are offset to have similar absorbance at 440 nm; the dashed line signifies the position of 356 nm. The absorbance of MSC-360 (**b**) is subtracted by the value at the corresponding wavelength evolved at 1 min; the solid lines in **b** are for visual guide. Obviously, the production of CdS MSC-360 is suppressed upon increasing the CdCl_2_ concentrations.

To intensify the production of CdS MSC-360, we increase the concentration of the solution, the result of which is shown in Fig. 1a. Figure 3 presents the absorption spectra of four solutions with four different concentrations after 24 h of preparation (a), and a summary of time-dependent absorbance of CdS MSC-360 (at its lowest energy transition peak position) in these solutions for times up to 24 h (b). Mixtures with the same feed molar ratio as that used in Fig. 1a are placed in the same solvent of BTA (1.5 mL) and water (1.5 mL), but with resulting CdCl_2_ concentrations of 2.0 (magenta trace (a) and circular symbols (b)), 4.0 (blue trace (a) and square symbols (b)), 6.0 (green trace (a) and triangular symbols (b)), and 10.0 mM (gray trace (a) and diamond symbols (b)). The solutions are kept in cuvettes for the absorption measurements after different time durations.

The solution with 2.0 mM CdCl_2_ effectively produces CdS MSC-360, with the absorbance of CdS MSC-360 increasing gradually to about 0.42 after 24 h. When the concentration of CdCl_2_ is increased to 4.0 mM, the absorbance of MSC-360 at 24 h is reduced to 0.31. With 6.0 mM of CdCl_2_, the strength of MSC-360 at 24 h is further reduced to 0.25. When the concentration of CdCl_2_ is increased to 10.0 mM, an evolution of MSC-360 does not occur. Accordingly, an increase in the solution concentration suppresses the production of CdS MSC-360.

For the two solutions of 2CdCl_2_-8MPA-20KOH-1TU in the BTA (1.5 mL) and water (1.5 mL) solvent with the CdCl_2_ concentrations of 5.0 and 10.0 mM, dynamic light scattering (DLS) (Supplementary Fig. 12) indicates large aggregates are present with hydrodynamic diameters (*D*_h_) of 272 ± 4 and 379 ± 3 nm, respectively. The higher the solution concentration is, the larger the aggregates are. We would like to point out that these aggregates are much larger than those which are resulted from the self-assembly of Cd and S precursors in the organic-phase reactions^18–20,28^. The formation of such large aggregates has also been validated by TEM (Supplementary Fig. 13).

To investigate why the aggregation occurs, we use the fluorescence spectroscopy of pyrene. This molecule has been used as a solvatochromic probe for aqueous solutions to detect the onset of self-assembly and/or aggregation, which can result in moieties including micelles and nanofibers^44–48^. Pyrene has high affinity for nonpolar environments, such that when pyrene moves into a relatively more hydrophobic environment, the fluorescence intensity ratio of its third (I_3_) to first (I_1_) peaks increases. Supplementary Fig. 14 presents the I_3_/I_1_ ratios of pyrene for two types of mixtures, which are 2CdCl_2_-8MPA-20KOH (a) and 2CdCl_2_-8MPA-20KOH-1TU (b). The mixtures are placed in 3.0 mL of water and in the BTA (1.5 mL) plus water (1.5 mL) solvent, with different CdCl_2_ concentrations ranging from 0.5 to 80.0 mM. When the CdCl_2_ concentration is higher than 4.0 mM, the I_3_/I_1_ ratio increases significantly for the two types of mixtures without or with TU. From these observations, the critical aggregation concentration (CAC) of CdCl_2_ can be estimated to be ~4.0 mM. The term “Cd–MPA complex” was used for the aqueous-phase approach to CdTe QDs with a reaction of CdCl_2_, MPA, NaOH, and Te precursor in water^34,36^.

Accordingly, we hypothesize that the “Cd–MPA complex” forms in our 2CdCl_2_-8MPA-20KOH solutions and aggregates when the CdCl_2_ concentration is higher than the CAC. In our 2CdCl_2_-8MPA-20KOH-1TU solutions, when the CdCl_2_ concentration is lower than CAC, both the Cd–MPA complex and TU are present in the BTA-containing aqueous environment, and CdS MSC-360 evolves readily (as presented in Fig. 1a). When the CdCl_2_ concentration is higher than the CAC, it is the Cd–MPA complex that dominates the aggregation observed. The resulting aggregates effectively separate a majority of the Cd–MPA complex from the BTA molecule which stays in the solution phase (outside of the aggregates). Consequently, the production of CdS MSC-360 is suppressed almost completely when the CdCl_2_ concentration is as high as 10.0 mM (Fig. 3 gray trace and diamond symbols). Thus, a relatively low CdCl_2_ concentration of 2.0 mM is used in this study to synthesize CdS MSC-360 with a constant Cd to MPA feed molar ratio of 1 to 4. In the present work we also discovered that when the ratio was 1 to 1, precipitation took place, and when the ratio was 1 to 2, the CAC became larger at about 20.0 mM.

## Discussion

**Fig. 4:**
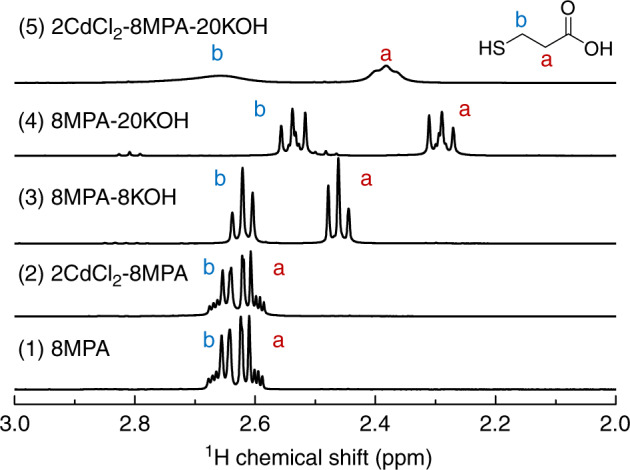
^1^H NMR spectra of five MPA-containing solutions in D_2_O. The MPA concentration is 40.0 mM for the five solutions, which consist of 8MPA (1), 2CdCl_2_-8MPA (2), 8MPA-8KOH (3), 8MPA-20KOH (4), and 2CdCl_2_-8MPA-20KOH (5). The feed molar ratio indicated follows that of the mixture studied in Fig. 1, while the CdCl_2_ concentration in Solutions 2 and 5 is 10.0 mM. For the MPA molecule, the two protons of the two methylene groups are labeled as a and b. Evidently, the formation of the Cd–MPA complex involves the deprotonation of the thiol group. Compared to trace 4, the chemical shift and broadening of the two resonance signals in trace 5 is indicative of the formation and aggregation of the Cd–MPA complex, which is in agreement with the ^13^C NMR and pyrene results shown in Supplementary Figs. 15 and 14 for the corresponding solutions, respectively.

For the Cd–MPA complex that has been suggested to be present in the basic environment^34,36^, there has been no information available regarding their formation and aggregation. To have a fundamental understanding of such processes, we use NMR spectroscopy to study five MPA-containing solutions in D_2_O with an MPA concentration of 40.0 mM. Figure 4 shows the ^1^H NMR spectra of 8MPA (1), 2CdCl_2_-8MPA (2), 8MPA-8KOH (3), 8MPA-20KOH (4), and 2CdCl_2_-8MPA-20KOH (5). For Solutions 2 and 5, the CdCl_2_ concentration is 10.0 mM. Supplementary Fig. 15 displays the corresponding ^13^C NMR spectra of these five solutions. All the spectra are collected at room temperature. For the MPA molecule, HOOC–CH_2_–CH_2_–SH, the two methylene groups are referred to a and b, which are next to the carboxyl and thiol groups, respectively.

The ^1^H NMR spectrum of MPA alone shows a resonance multiplet signal at about 2.63 ppm (trace 1), which changes little when CdCl_2_ is present (trace 2). For Solution 3 with the same KOH concentration as that of MPA (trace 3), two triplet resonance signals are observed. One triplet is located at 2.62 ppm (Proton b), while the other is at 2.46 ppm (Proton a). For Solution 4 with a KOH concentration 2.5 times of that of MPA (trace 4), the two triplet resonance signals are observed at 2.54 (proton b) and 2.29 ppm (proton a). For Solution 5 (trace 5) upon the presence of CdCl_2_, two broad resonance signals appear at 2.66 (proton b) and 2.38 ppm (proton a). It is noteworthy that the former resonance signal is broader than the latter.

From Solutions 1, 3–4, the upfield shift and separation of the two triplets is related to the deprotonation of the thiol and carboxyl groups in the course of the increase of the pH. Taking account of the pK_a_ values of carboxyl (4.3) and thiol (10.8) groups in MPA^49^, it is reasonable that the two functional groups remain protonated in Solution 1 (pH ~2.6). In Solution 3 (pH ~5.2), the carboxyl group is deprotonated, while the thiol group is not. In Solution 4 (pH ~11.9), the two groups are deprotonated. From Solutions 4–5, the resonance signals display downfield shifts, suggesting the formation of the Cd–MPA complex. For the formation of Cd-based complexes, such as Cd–TOP and Cd–TOPO complexes, downfield shifts have been observed by ^31^P NMR^50,51^. TOP and TOPO represent tri-*n*-octylphosphine and tri-*n*-octylphosphine oxide, respectively. It is noteworthy that after the addition of CdCl_2_ to Solution 3, precipitation happens. Furthermore, due to the protonation of both the carboxyl and thiol groups in Solution 1, there is no obvious change of the ^1^H NMR spectra in the presence of CdCl_2_ (trace 2).

The ^1^H NMR results demonstrate that CdCl_2_ and MPA have no coordination interaction in an acidic environment (pH ~3.0), while the Cd–MPA complex forms in a basic environment (pH ~12.0) when the carboxyl and thiol groups are deprotonated. It has been known that NMR signal linewidth is inversely related to the spin-spin relaxation time (T_2_). Signal broadening suggests reduced T_2_, which may result from slower motion of larger aggregates with enhanced masses^22,50^. Therefore, the broadening of the two resonance signals of Solution 5 is indicative of the aggregation of the Cd–MPA complex. While Proton b shows the broadening more with a weaker feature than Proton a does, it is reasonable to conclude that the Cd^2+^ ion coordinates with the deprotonated thiol group for the formation of the Cd–MPA complex. The formation and aggregation of the Cd–MPA complex is also supported by the corresponding ^13^C NMR of MPA in the five solutions (Supplementary Fig. 15). We add TU into Sample 5 (Supplementary Fig. 16); the ^1^H and ^13^C NMR measurements suggest further that the aggregation is driven by the Cd–MPA complex.

**Fig. 5:**
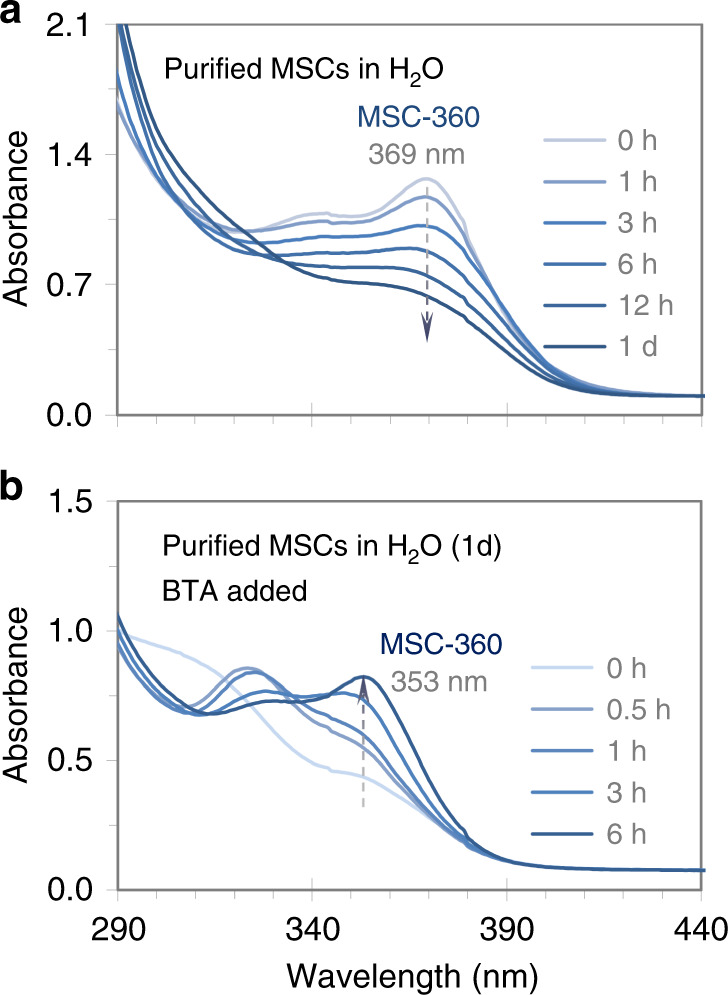
Absorption spectra of an aqueous dispersion of purified CdS MSC-360 before and after mixing with BTA. All spectra are offset to have similar absorbance at 440 nm. Purified CdS MSC-360 is dispersed in deionized water with a concentration of about 1.0 g ∙ L^−1^, and the absorption measurements last for 1 day at various durations indicated **a**. CdS MSC-360 disappears gradually. Afterwards, 1.5 mL of the dispersion is mixed with BTA (1.5 mL) for additional measurements up to 6 h **b**. CdS MSC-360 evolves again. In water (**a**), CdS MSC-360 transforms to its PC; upon the presence of BTA (**b**), the PC to MSC-360 transformation occurs, with almost complete recovery of MSC-360 in the relatively short period of 6 h.

In organic-phase syntheses, the formation pathway of binary colloidal semiconductor ME MSCs comprises an intramolecular reorganization of their particular PC molecules^18–28^. Whether the aqueous-phase MSCs follow the same pathway of formation is an important question. Figure 5 presents the absorption spectra collected at various elapsed time durations up to 1 day after a CdS MSC-360 purified sample is dispersed in deionized water at room temperature (a), with the concentration of MSC-360 estimated about 1.0 g ∙ L^−1^. After 1 day, 1.5 mL of the dispersion is mixed with 1.5 mL of BTA, and optical absorption measurements are performed at different periods up to 6 h (b).

Figure 5 suggests that such intramolecular reorganization remains important in the formation of the aqueous-phase CdS MSC-360. In Fig. 5a, the dispersion initially exhibits an absorption peaking at 369 nm with an optical density of ~1.3. In the following 1 day, the amount of MSC-360 decreases gradually. After 1 day, the absorbance at 369 nm decreases to ~0.6. Further decrease is observed up to 4 days (Supplementary Fig. 17a). In Fig. 5b, the absorption peaking at ~353 nm keeps increasing, and becomes the major peak after 6 h. These results suggest that the recovery of MSC-360 appears almost complete upon the presence of BTA (with the dilution). When deionized water is used instead of BTA (Supplementary Fig. 17b), a recovery of CdS MSC-360 is not observed. With a smaller amount of BTA (Supplementary Fig. 18) and with different primary amines such as PrA and ETA (Supplementary Fig. 19), a recovery of MSC-360 can also be obtained. We would like to point out that since the peak position of the lowest energy absorption for the present MSCs varies significantly with solvent from 369 nm in deionized water to 353 nm in the BTA and water mixture, a label of MSC-360 would seem reasonable for these CdS MSCs.

In water (Fig. 5a), the disappearance of CdS MSC-360 indicates that an MSC to PC transformation has happened. Upon the presence of a primary amine (Fig. 5b), the process of the CdS MSC-360 recovery is quite similar to the PC to MSC transformation that occurs upon the presence of a primary amine such as BTA or octylamine (OTA) for two-step approaches which are carried out in nonaqueous environments, such as CdSe, and CdTe MSCs in toluene^21–24^, and ZnSe MSCs in cyclohexane^27^. The reversible transition process may be related to changes in surface passivation^26^. Close examination of panels a and b in Fig. 1 suggests that it is reasonable to infer that the consumption of the reactants (which resulted in the formation of the MSC PC) took place mainly in the first 6 h, while the PC to MSC-360 transformation (via intramolecular organization) continued for up to 12 h (shown). The relatively fast PC formation compared to the PC transformation to MSC-360 supports that the ~60% conversion yield was estimated for the PC formation (Supplementary Fig. 2). The different rates between the PC formation and PC transformation to MSCs can be found elsewhere^25,52^.

In conclusion, we have developed an effective aqueous-phase approach to CdS MSC-360 at room temperature and have explored the formation pathway. CdCl_2_ and TU are used as the respective Cd and S sources, with MPA as the ligand. For the synthesis, a mixture with the feed molar ratio of 2CdCl_2_-8MPA-20KOH-1TU is prepared; the mixture is then added to deionized water followed by the addition of BTA. The CdCl_2_ concentration in the resulting solution is 2.0 mM. The presence of BTA is a prerequisite for the room-temperature evolution of CdS MSC-360 (Fig. 1). While the amount of BTA matters, other primary amines such as PrA and ETA also promote the room-temperature evolution of MSC-360 (Fig. 2). The primary amine assists the room-temperature decomposition of TU in the presence of CdCl_2_ (Supplementary Fig. 10). Furthermore, the CdCl_2_ concentration matters (Fig. 3), due to the possible aggregation with the CAC of ~4.0 mM. Larger CdCl_2_ concentration give rise to larger aggregates. We argue that a Cd–MPA complex forms in a 2CdCl_2_-8MPA-20KOH solution with a basic environment (pH = ~12), in which the thiol and carboxyl groups of MPA are both deprotonated and a coordination interaction occurs between the former group and CdCl_2_ (Fig. 4). The comprehensive identification of the aggregation, which is dominated by the Cd–MPA complex with the CAC of ~4.0 mM, supports that the mechanism of the nucleation and growth of QDs is more complicated in aqueous-phase syntheses than in organic-phase ones^30,37^. We propose that the reactivity of TU is enhanced via the coordination with the Cd–MPA complex, followed by the BTA-assisted decomposition of the activated TU, which facilitates the room-temperature formation of Cd–S covalent bonds. Supplementary Fig. 20 depicts the possible reactions that could be involved in the BTA-assisted decomposition of TU, similar to those hypothesized for the BTA-assisted decomposition of diphenyl TU in DMF^43^. In deionized water, CdS MSC-360 (purified) disappears gradually; however, when BTA is introduced, the population of CdS MSC-360 recovers (Fig. 5). Thus, the formation pathway of aqueous-phase MSCs follows the PC to MSC transformation, similar to that in the organic-phase evolution^18–28^. The findings introduce a room-temperature aqueous-phase approach to forming semiconductor CdS MSC-360. As always, it is impossible to have a complete understanding of the formation mechanism from one study. The formation and aggregation of the Cd–MPA complex in basic aqueous environments have been quite unexpected and should be conceptually helpful even when other ligands are used. For the approach developed, we are actively exploring its applicability for the synthesis of other II–VI semiconductor MSCs, such as those of CdSe and CdTe. The present study brings deeper insights for the formation pathway of colloidal semiconductor MSCs, conveying comprehension into the pre-nucleation stage and contributing to the advance of the nanocrystal synthesis from an empirical art to science.

## Methods

### Chemicals

MPA (99%), BTA (99%), ETA aqueous solution (ETA, 70%), deuterium oxide (D_2_O, 99%), and pyrene (99%) were purchased from Aldrich. CdCl_2_·2.5H_2_O (99%) and TU (99%) were purchased from Chengdu Kelong Chemical. Ethanol (99%) was from Chengdu Haixin Chemical. Potassium hydroxide (KOH, 99%) and propylamine (PrA, 99%) were obtained from Tianjin Zhiyuan Chemical. All the chemicals are used without purification (except stated otherwise).

### Preparation of Cd and S precursor stock solutions and CdS MSCs

A Cd precursor stock solution was prepared by dissolving CdCl_2_·2.5H_2_O (0.1827 g, 0.8 mmol) in deionized water (5.0 mL) with MPA (279 μL, 1.6 mmol), to which KOH solution (5 M, about 1.6 mL) was added to adjust the pH to about 11.7. Additional deionized water was added to make the total volume of 10.0 mL, with the pH changed little. A S precursor stock solution was obtained by dissolving TU (0.0304 g, 0.4 mmol) in deionized water (10.0 mL). For the room-temperature aqueous-phase synthesis of CdS MSCs (such as shown in Fig. 1a), 75 μL Cd precursor solution and 75 μL S precursor solution were mixed together; the resulting mixture was added into 1.35 mL deionized water, followed by the addition of 1.5 mL BTA. It is noteworthy that the aqueous solution has apparent pH values of 11.0 and 13.0 before and after the addition of BTA. The resulting solution consisting of CdCl_2_ (2.0 mM), TU (1.0 mM), and MPA (8.0 mM) was then allowed to react at room temperature for various durations. The colloidal reaction system remains stable up to 3 days.

### Purification of CdS MSCs

The as-synthesized CdS MSC aqueous sample (3.0 mL) was precipitated upon the addition of ethanol (9.0–12.0 mL). The resulting turbid solution was then centrifuged on a Beckman Coulter Allegra 64R at room temperature with a speed of 8000 rpm for about 2 min. After centrifugation, the resulting precipitate was collected and evacuated for 1 h to remove the solvent residual.

### Ultraviolet absorption spectroscopy

Absorption spectra were collected using a TECHCOMP UV 2310 II ultraviolet–visible (UV–vis) absorption spectrometer, with a 1-nm data collection interval. A cuvette with deionized water was used as a background sample.

### Photoluminescence spectroscopy

Pyrene (0.3 μM) was used as a probe to investigate the aggregation. A solution of pyrene was prepared in water or the mixture of BTA and water with equal volumes, to which a mixture of 2CdCl_2_-8MPA-20KOH-1TU was added with various CdCl_2_ concentrations. The samples prepared in deionized water were kept at room temperature for 1 day before emission measurements. Those prepared in the mixture of BTA and water were stored for about 10 min only before measurements; longer storage would consume the precursors and form CdS MSCs. The photoluminescence spectra were recorded with an excitation wavelength of 335 nm and with both excitation and emission slit widths of 2.5 nm. For the pyrene spectra, the intensity of the third (I_3_, at about 380 nm) and the first peaks (I_1_, at about 370 nm) was extracted, and the I_3_/I_1_ ratio was then plotted vs the CdCl_2_ concentrations.

### Dynamic light scattering (DLS)

DLS measurements were carried out on a Zetasizer Nano ZS90 instrument. The two 2CdCl_2_-8MPA-20KOH-1TU samples were placed in the solvent of BTA (1.5 mL) and water (1.5 mL). Each sample was kept for about 10 min at room temperature before measurement; longer storage would consume the precursors and form CdS MSCs.

### ^1^H and ^13^C NMR spectroscopy

The samples for both ^1^H and ^13^C NMR measurements were prepared in D_2_O. Each sample was kept for about 2 h at room temperature before measurement. The NMR spectra were recorded on Bruker Avance II spectrometers. The ^1^H NMR spectra were corrected with the characteristic resonance signals of the solvent D_2_O. The ^1^H and ^13^C NMR spectra were collected at 400 and 200 MHz, respectively.

## Supplementary information

Supplementary Information

## Data Availability

The authors declare that all relevant data supporting the findings of this study are available from the authors on request.
