# Supplementary material for: Room-temperature formation of CdS magic-size clusters in aqueous solutions assisted by primary amines
Source: Nat Commun. 2020 Aug 21;11:4199. doi: 10.1038/s41467-020-18014-6 (PMC7442802; doi:10.1038/s41467-020-18014-6)
Supplement: Supplementary file 1 — Supplementary Information [file 41467_2020_18014_MOESM1_ESM.pdf]

Supplementary Information

Room-temperature formation of CdS magic-size clusters in aqueous solutions assisted by primary amines

Yu* et al.

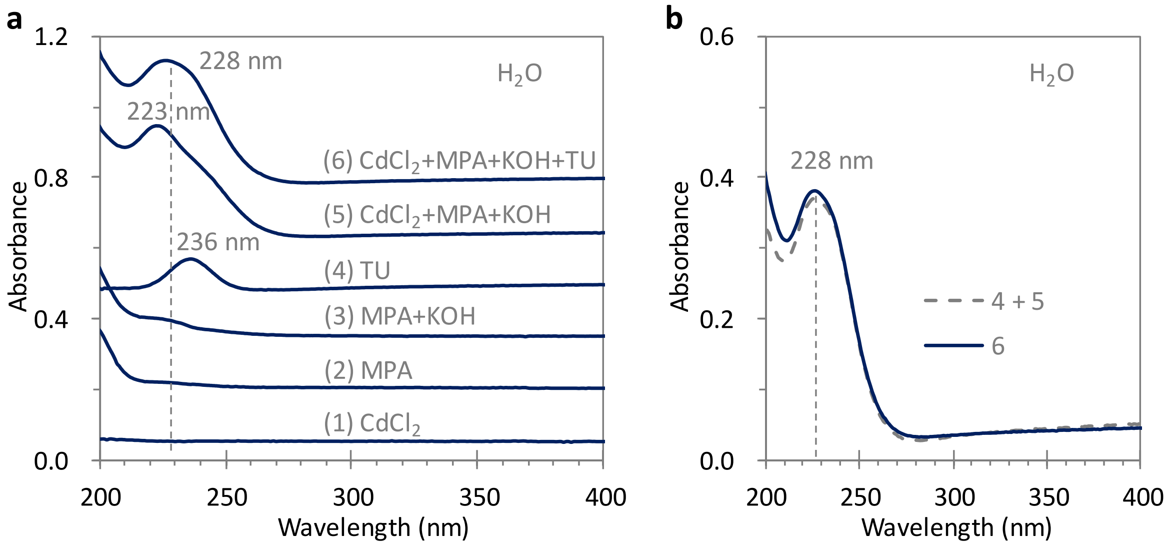

Supplementary Figure 1. (a) Absorption spectra of aqueous solutions of 20 µM $CdCl_2$ (1), 80 µM MPA (2), 80 µM MPA with a pH about 11.5 (3), 10 µM TU (4), and a mixture of 20 µM $CdCl_2$ and 80 µM MPA with a pH about 11.5 without (5) and with (6) 10 µM TU. Deionized water is used, with KOH controlling the pH value. (b) Absorption comparison of Sample 6 (solid trace) and the superimposed absorption of Samples 4 and 5 (dashed trace). The two vertical dashed lines in a and b indicate the position of 228 nm. Evidently, the absorption of Sample 6 is similar to the sum of the absorption of Samples 4 and 5, peaking at ~228 nm.

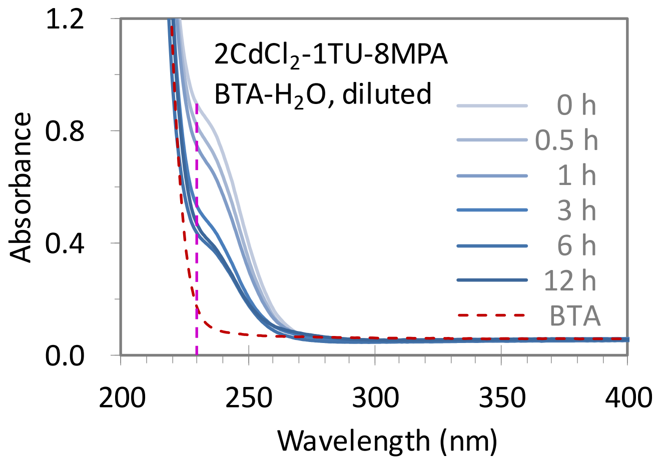

Supplementary Figure 2. Absorption spectra used to estimate the conversion yield. This modified Fig. 1b includes additional UV absorption behavior of a BTA solution (84.3 mM, red dashed curve). This solution was made with 25 µL of BTA in 3.0 mL of water. The dashed vertical line highlights the wavelength at 230 nm. Based on the decrease at 228 nm of the UV absorption, the overall conversion yield is estimated to be about 61% after a period of 6 h; from 6 to 12 h, there was not much more reaction going on. In a side note, the overall conversion yield (based on the decrease at 230 nm in absorbance) is estimated to be about 60% after a period of 6 h. It is reasonable to infer that the consumption of reactants (which resulted in the formation of the MSC precursor compound (PC)) took place mainly in the first 6 h, while the PC to MSC-360 transformation (via intra-molecular organization) continued for up to 12 h. Reasonably, the PC formation is faster than the PC to MSC-360 transformation, and the 60% conversion yield estimated is related to the former.

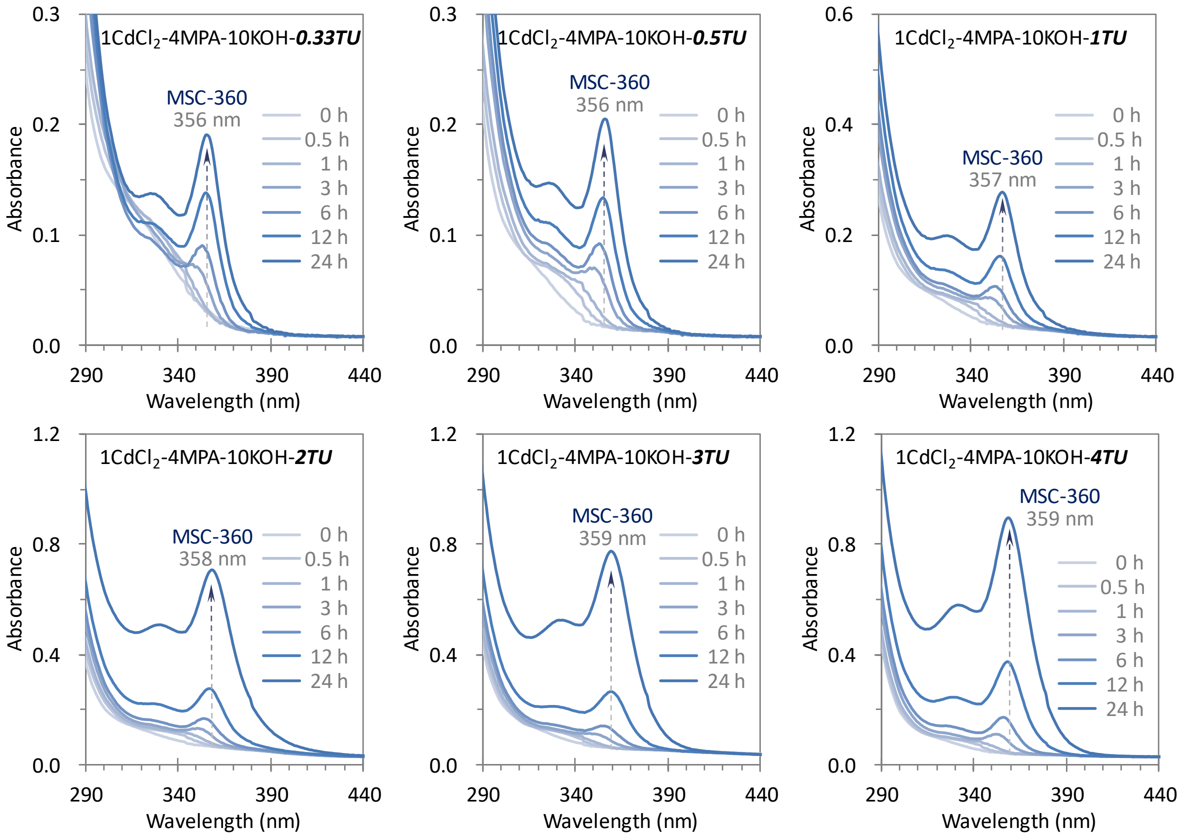

Supplementary Figure 3. Time-resolved optical absorption spectra collected from six reactions with various Cd-TU feed molar ratios as indicated (under a constant Cd concentration of 2.0 mM). For each reaction, the solvent is the mixture of BTA (1.5 mL) and water (1.5 mL). Each reaction occurs in a cuvette which is used directly for the spectroscopy study. The spectra of each reaction have been offset to have the same absorbance value at 440 nm. The dashed arrows signify the wavelength positions indicated. Clearly, the characteristic optical absorption peak of MSC-360 broadens gradually upon increasing the TU concentration. In this regards we use the 2$CdCl_2$ to 1TU feed molar ratio to synthesize CdS MSC-360. The more TU is used, the more CdS MSCs are produced, suggesting that the decomposition of TU might be a rate-determination step.

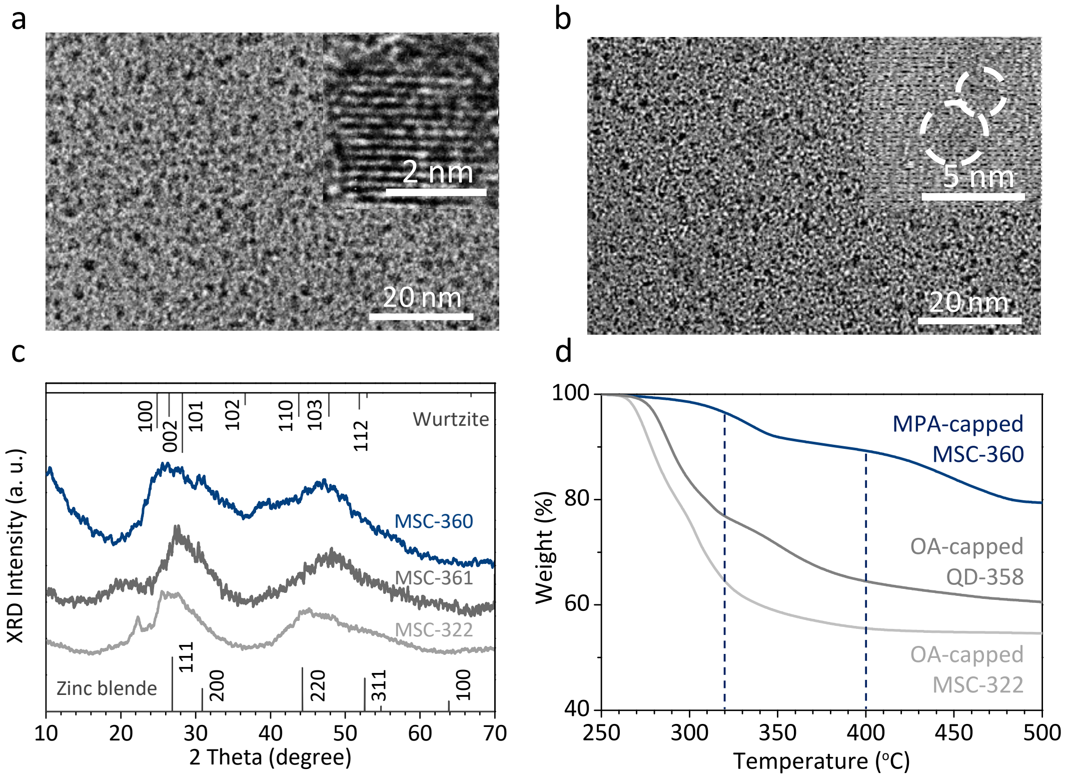

Supplementary Figure 4. Transmission electron microscopy (TEM) typical images of as-synthesized (a) and purified (b) MPA-capped CdS MSC-360, powder X-ray diffraction (XRD) patterns (c), and thermogravimetric analysis (TGA) (d) of purified MPA-capped CdS MSC-360. The CdS MSC-360 is obtained from the reaction of $CdCl_2$ (2.0 mM), MPA (8.0 mM), KOH (20.0 mM), and TU (1.0 mM) in a mixture of BTA (1.5 mL) and water (1.5 mL). See Supplementary Note 1 for additional information.

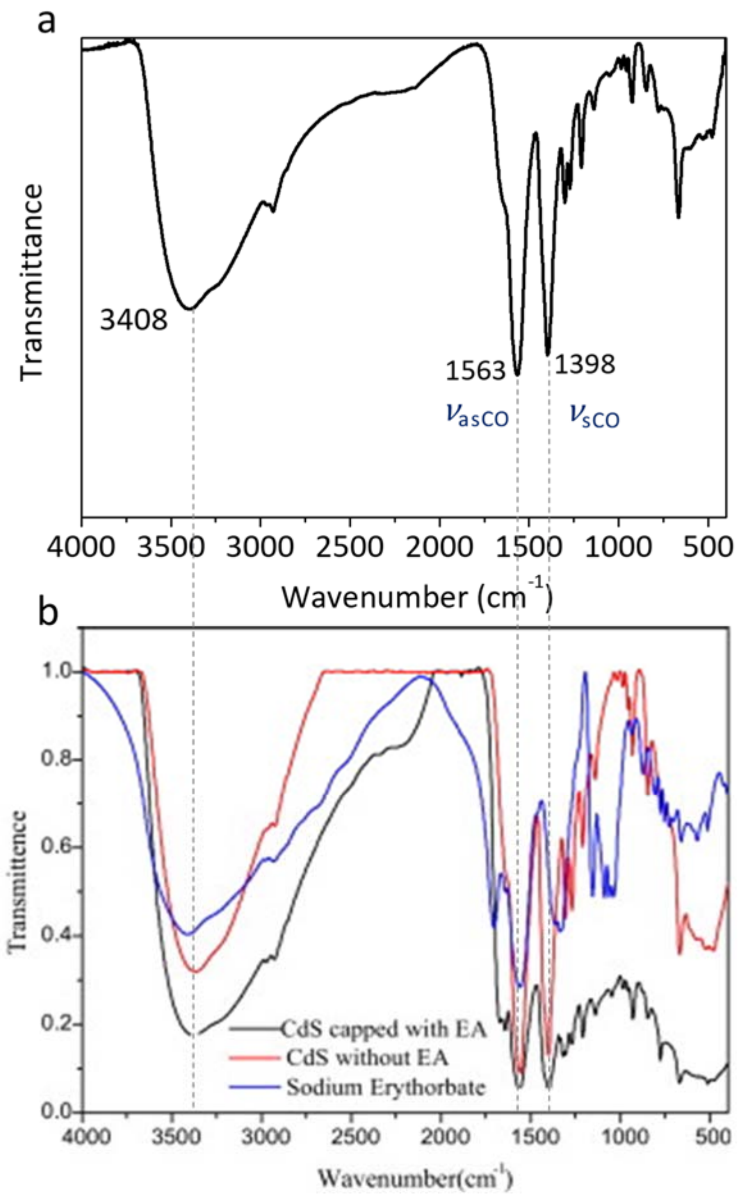

Supplementary Figure 5. Comparison of Fourier Transform Infrared (FTIR) spectra for various materials, namely purified MPA-capped CdS MSC-360 (a) and MPA-capped CdS QDs (b, red trace). The three spectra shown in Part b are published results[1]. For the samples of Part b, the MPA-capped CdS QDs (red trace) were synthesized in an aqueous phase with $CdCl_2$, Na_2S, MPA and NaOH; in the presence of erythorbic acid (EA, 99%, Aladdin Chemistry Co. LTD), the resulting QDs (labelled "capped with EA") are represented by the black trace. The blue trace is from sodium erythorbate which was made from EA and NaOH. The MSC-360 and two QD samples have similar FTIR spectra, with respect to two characteristic FTIR peaks at 1563 and 1398 cm^{-1}. These two frequencies are respectively due to the characteristic asymmetric (ν_{asCO}) and symmetric (ν_{sCO}) C-O stretching vibration bands of the carboxylate

moiety (-COO^-) of the MPA ligand. The FTIR feature at approximately 3408 cm^{-1} can be attributed to moisture which was apparently present in the samples[2]. These three peaks are indicated by three vertical dashed lines. Importantly, the MPA-capped MSC-360 sample (a) does not exhibit any of the FTIR characteristic peaks of BTA[3], indicating that this sample does not contain BTA molecules and its surface ligands are MPA only. For the sample of CdS nanocrystals which were passivated by BTA molecules[3], its FTIR spectrum contained three distinct IR absorption features at frequencies associated with the N–H bond, which are at 3300 cm^{-1} (a doublet feature, corresponding to the stretching vibration), 1573 cm^{-1} (a singlet feature, due to the bending vibration), and 850 cm^{-1} (attributed to the out-of-plane bending vibration)[3]. It is noteworthy that the resolutions of the parts a and b are different. This is due to the fact that the former displays our own measurement, while the latter is copied and pasted from the literature[1]. (Adapted with permission from Ref 1. Copyright (2014) Elsevier.)

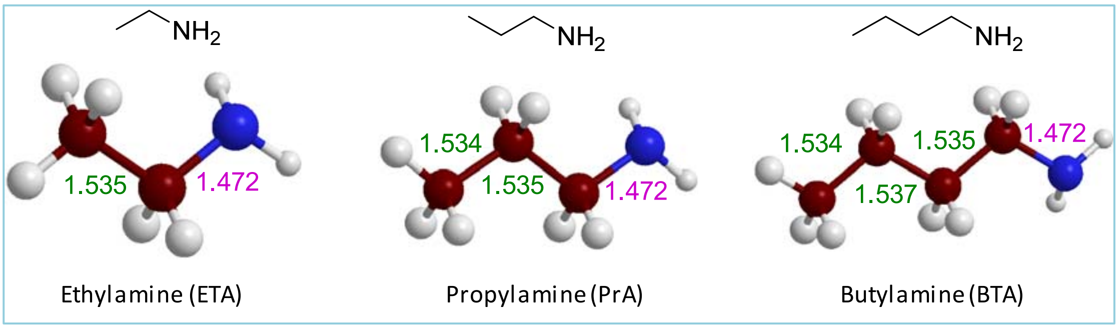

Supplementary Figure 6. Chemical structures and 3D models (by Chem3D) for the three primary amines that were used. These amines are ethylamine (ETA), propylamine (PrA), and butylamine (BTA). The different colors in these models represent H (white), C (red), and N (blue) atoms. The C-N and C-C bond lengths (Å) are presented with the color of purple and green, respectively. These two bond lengths are similar for the three primary amines.

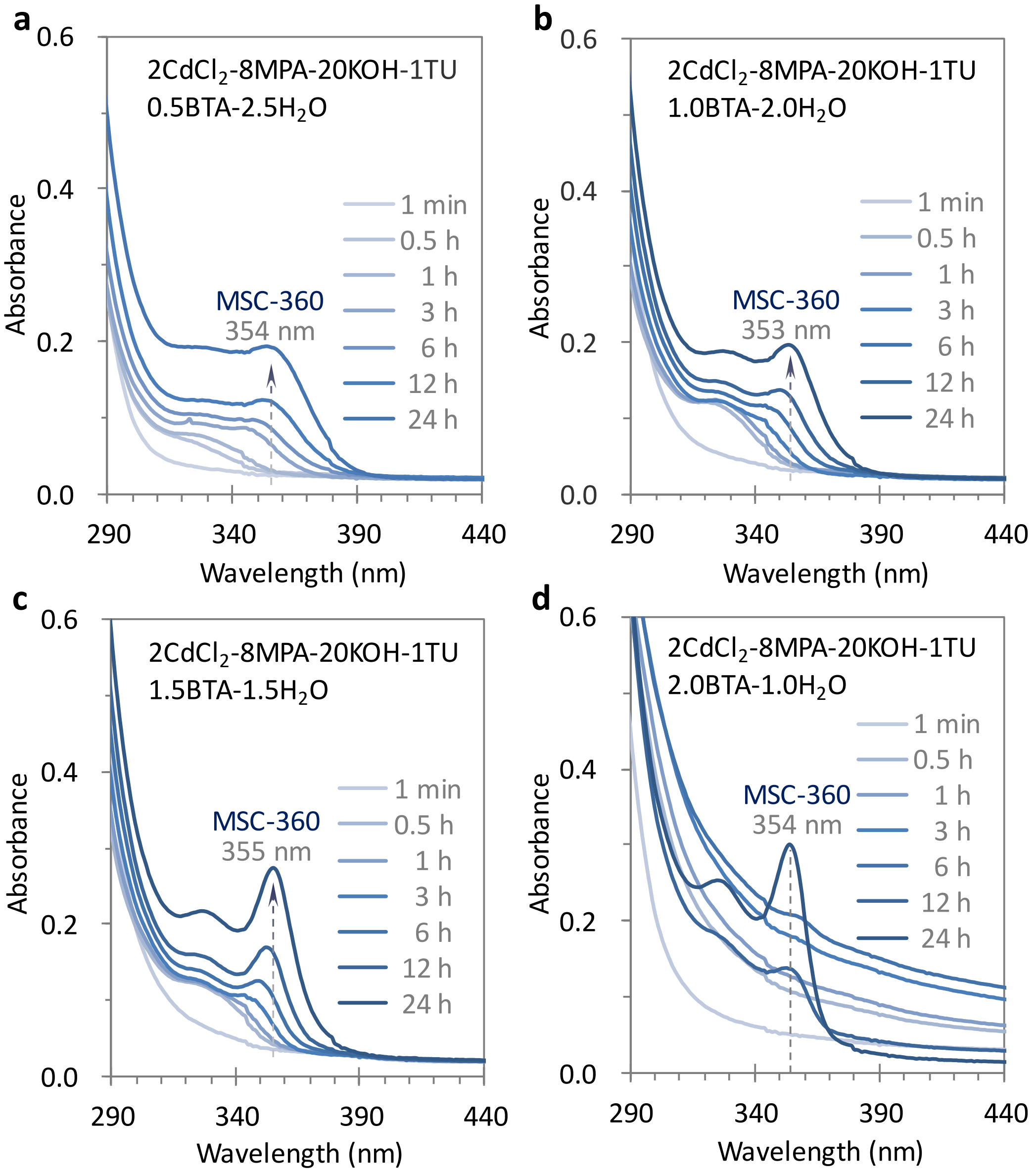

Supplementary Figure 7. Absorption spectra collected from the mixture of $CdCl_2$ (2.0 mM), MPA (8.0 mM), KOH (20.0 mM) and TU (1.0 mM), which is placed in the four BTA-water mixtures (3.0 mL) with the BTA volume of 0.5 (a), 1.0 (b), 1.5 (c), and 2.0 mL (d). The spectra in (a) to (c) are offset to have similar absorbance at 440 nm. The spectra in (d) are not offset to show the light scattering due to precipitation. The dashed arrows (a-c) and line (d) simplify the positions indicated. Evidently, CdS MSCs with a sharper absorption are produced at 24 h in a solvent with more BTA. It is noteworthy that for the mixture containing BTA (2.0 mL), the precipitate disappears after 6 h, with MSC-360 starting to evolve at around 12 h.

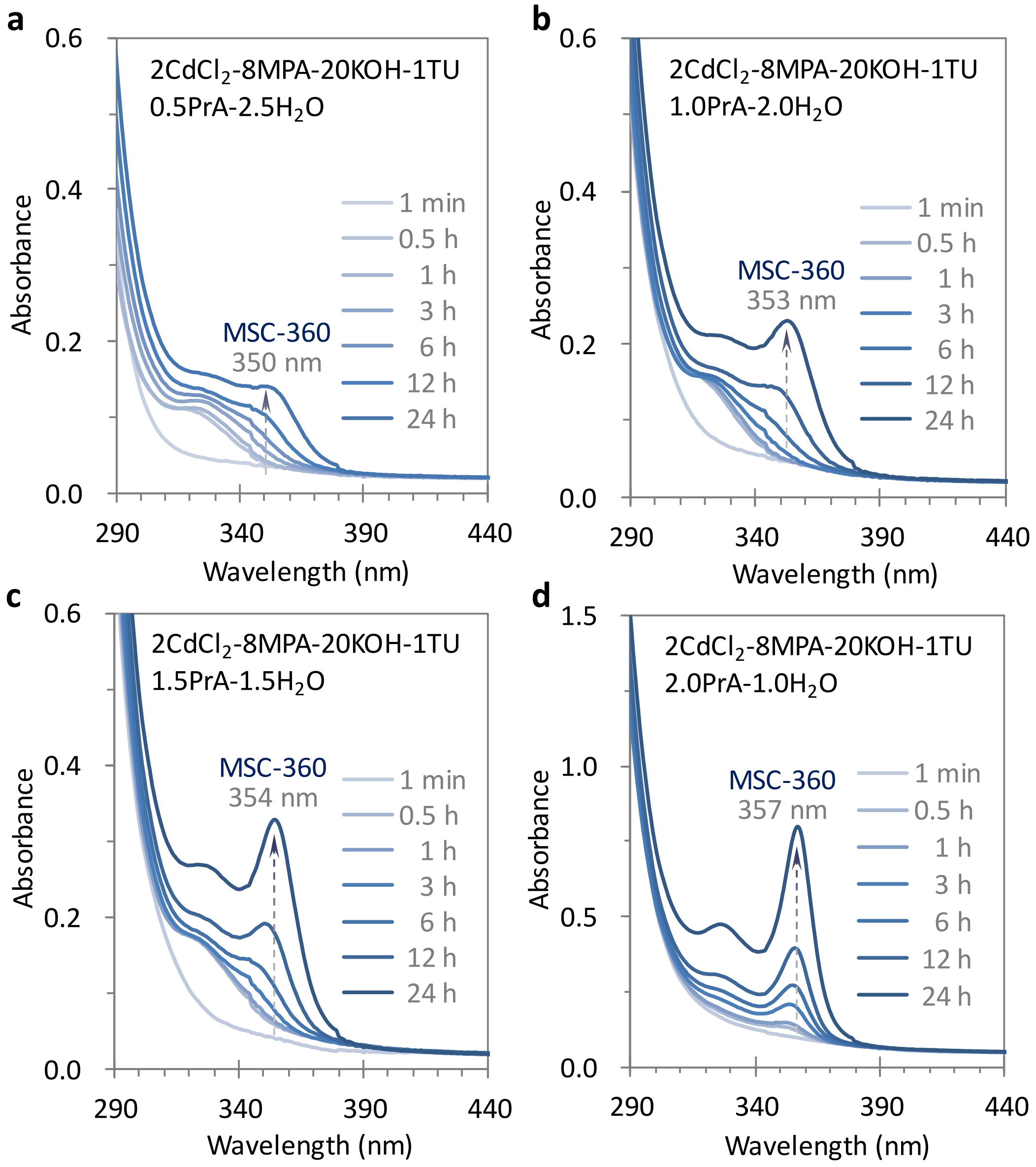

Supplementary Figure 8. Absorption spectra collected from the mixture of $CdCl_2$ (2.0 mM), MPA (8.0 mM), KOH (20.0 mM) and TU (1.0 mM), which is placed in the four 3.0 mL propylamine (PrA)-water mixtures. The volumes of PrA are 0.5 (a), 1.0 (b), 1.5 (c), and 2.0 mL (d). The spectra are offset to similar absorbance at 440 nm. The dashed arrows simplify the positions indicated. Evidently, CdS MSCs with a sharper absorption are produced at 24 h in a solvent with more PrA.

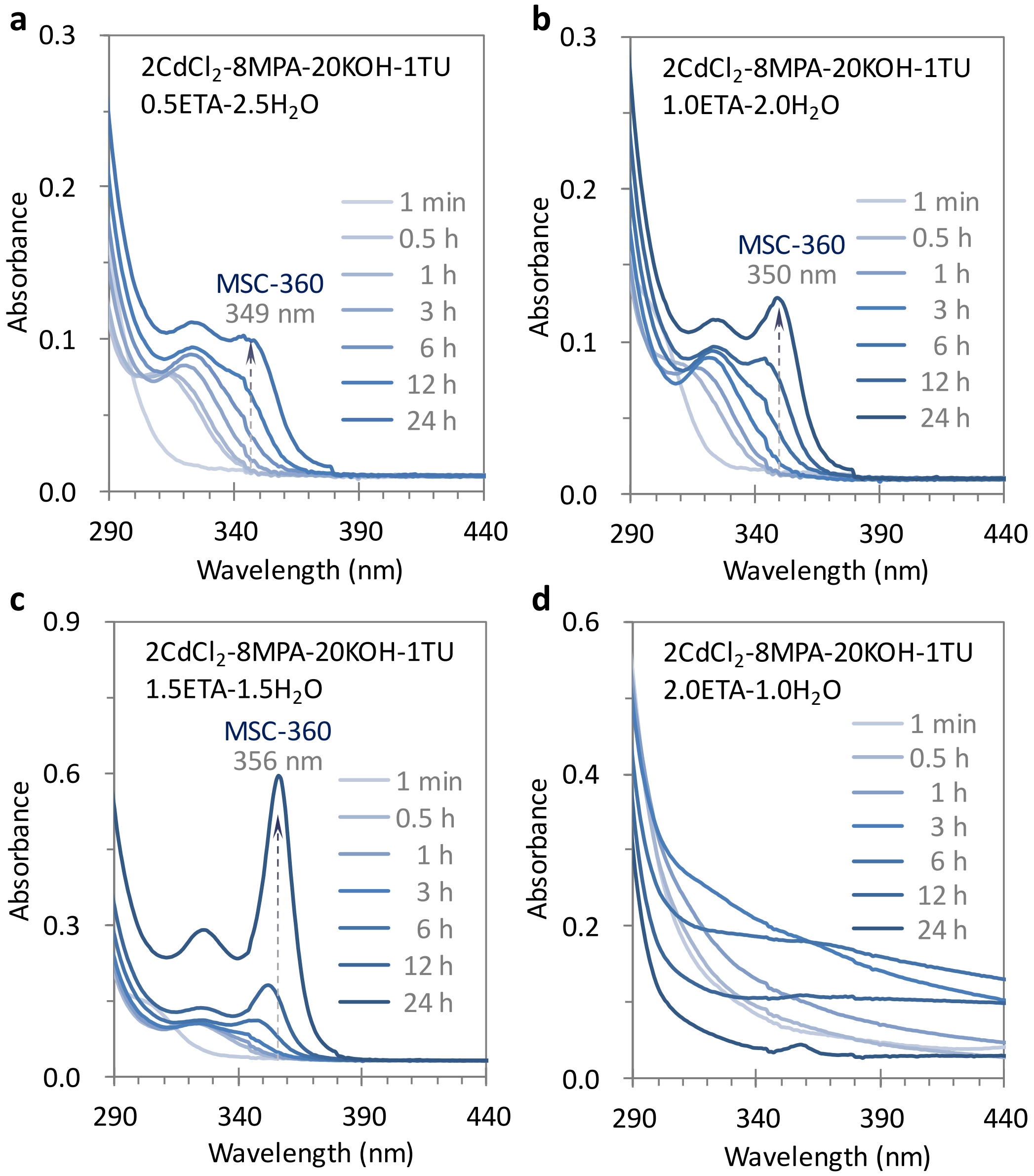

Supplementary Figure 9. Absorption spectra collected from the mixture of $CdCl_2$ (2.0 mM), MPA (8.0 mM), KOH (20.0 mM) and TU (1.0 mM), which is placed in the four 3.0 mL ethylamine (ETA)-water mixtures. The volumes of ETA are 0.5 (a), 1.0 (b), 1.5 (c), and 2.0 mL (d). Commercial ETA aqueous solution (70%) is used; thus, the practical volume of the ETA solution added is about 1.43 times to that labeled. The spectra in (a) to (c) are offset to have similar absorbance at 440 nm. The spectra in (d) are not offset to show the light scattering due to precipitation. The dashed arrows simplify the positions indicated. Evidently, CdS MSCs with a sharper absorption are produced at 24 h in a solvent with more ETA (1.5 mL). For the mixture containing ETA (2.0 mL), the precipitate almost disappears at 24 h (bottom trace).

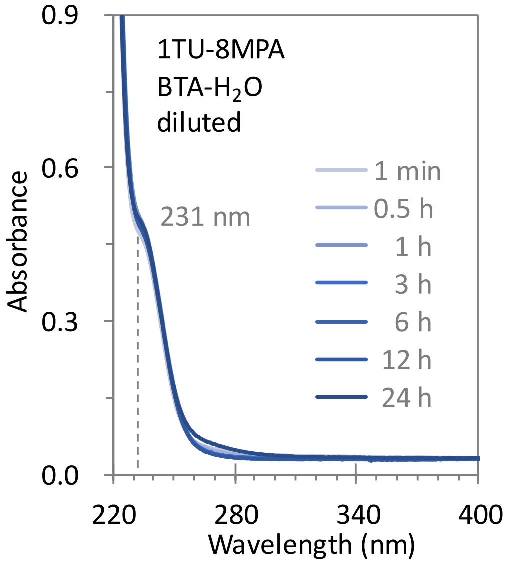

Supplementary Figure 10. Absorption spectra of a mixture of TU (1.0 mM) and MPA (8.0 mM) in a mixture of BTA (1.5 mL) and water (1.5 mL). After incubation for certain periods as indicated at room temperature, an aliquot (100 μL) of the solution is diluted in 3.0 mL of water for absorption measurements. The spectra are offset to the have similar absorbance at 400 nm. The dashed line signifies the position of 231 nm. The absorption spectra change little over time, suggesting that the TU remains stable.

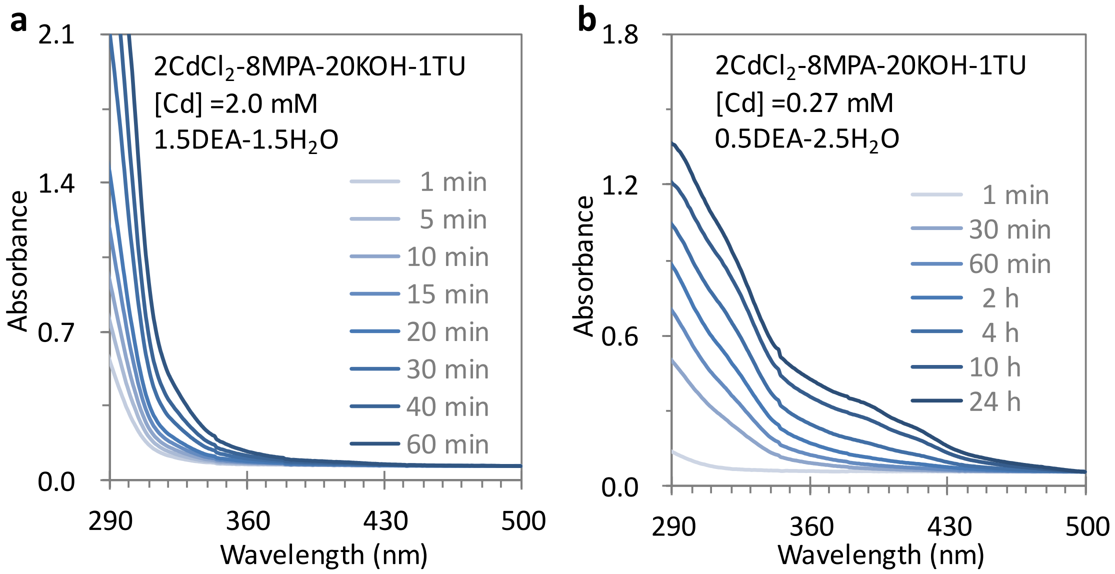

Supplementary Figure 11. Absorption spectra collected from two mixtures of $CdCl_2$ (2.0 mM), MPA (8.0 mM), KOH (20.0 mM) and TU (1.0 mM), with the concentrations of $CdCl_2$ of 2.0 (a) and 0.27 mM (b). The two mixtures are placed in 3.0 mL of mixture of diethylamine (DEA) and water, with the DEA volume of 1.5 (a) and 0.5 mL (b). Mixture a is prepared and measured at first; we notice that the optical intensity at low wavelengths (290 nm) increases significantly and becomes saturated after 60 min. Therefore, we decide to reduce the concentration of the mixture and the amount of DEA (b), to confirm that the optical intensity at the low wavelengths increases (which could be due to the formation of Cd–S covalent bonds[4]). The spectra are offset to have similar absorbance at 500 nm. Evidently, the secondary amine does not facilitate the evolution of CdS MSCs.

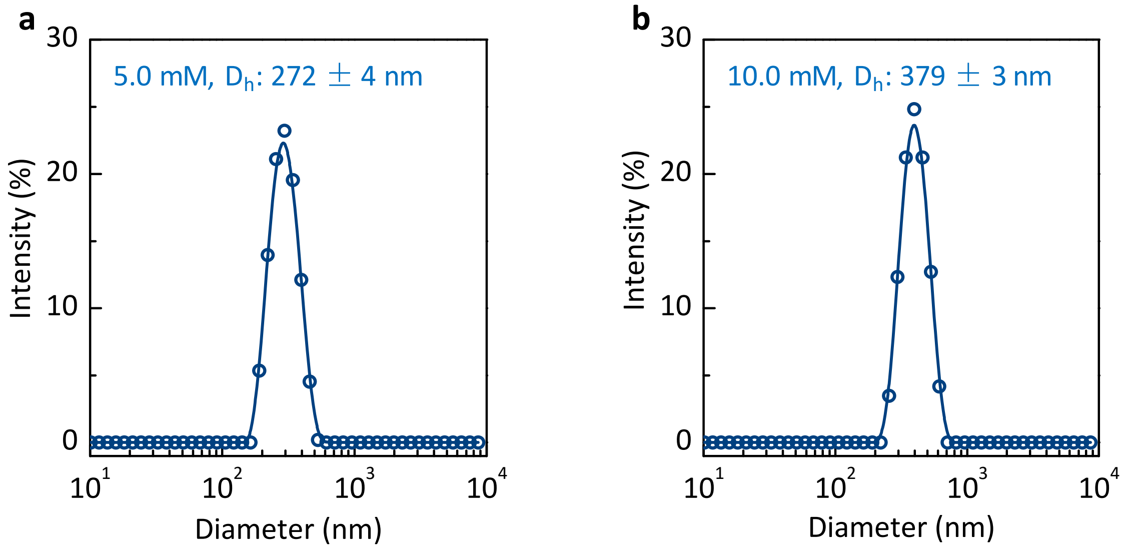

Supplementary Figure 12. Dynamic light scattering (DLS) patterns of two mixtures in the solvent of BTA (0.6 mL) and water (0.6 mL). The two mixtures have the same feed molar ratio of $2CdCl_2$-8MPA-20KOH-1TU, with the $CdCl_2$ concentration of 5.0 (a) and 10.0 mM (b). The DLS study indicates that aggregation occurs in the two solutions with the averaged aggregate diameter of 272 ± 4 (a) and 379 ± 3 (b) nm.

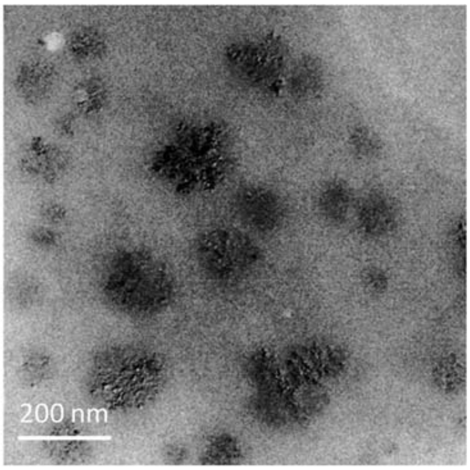

Supplementary Figure 13. A TEM image of one sample prepared from an aqueous solution of $2CdCl_2$ + 8MPA + 20KOH + 1TU with a $CdCl_2$ concentration of 40.0 mM. The TEM grid was prepared after storing the aqueous solution at room temperature for about 24 h. Aggregates with diameters ranging from 50 to 220 nm were observed. The TEM diameter is smaller than that measured with DLS due to the dehydration which occurs during the preparation of the TEM sample and during the measurement.

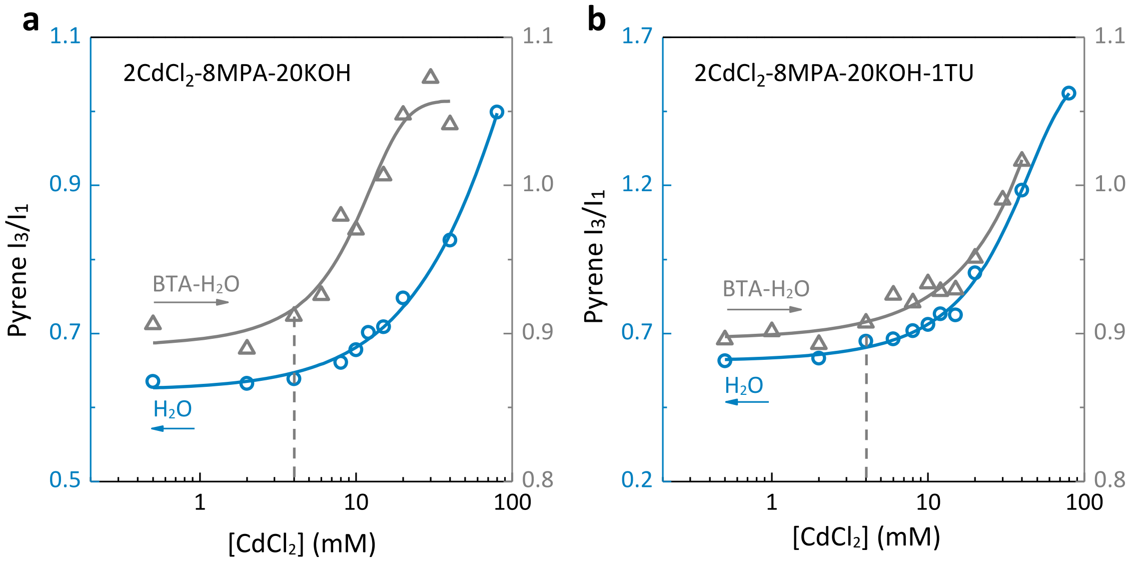

Supplementary Figure 14. The $CdCl_2$ concentration dependent I_3/I_1 ratios of pyrene (0.3 µM). Pyrene is added in the two solvents used, deionized water (blue circular symbols, left y axis) and a mixture BTA and water with equal volumes (grey triangular symbols, right y axis). The summary of the intensity ratio of the third peak (I_3, at about 380 nm) over the first one (I_1, at about 370 nm) is based on the emission spectra collected from two types of mixtures, 2Cd-8MPA-20KOH (a) and 2Cd-8MPA-20KOH-1TU (b). The resulting solutions have the $CdCl_2$ concentrations in the range from 0.5 to 80.0 mM. A stock solution with 80.0 mM $CdCl_2$ is prepared initially, and diluted to obtain the mixtures with the desired concentrations. The emission spectra of pyrene are obtained with an excitation wavelength of 335 nm. The measurements for water and the BTA-water solutions are performed, respectively, after 24 h and 10 min of preparation (at room temperature). The curves are drawn for visual guide. The critical aggregation concentration (CAC) is defined as the concentration at which the I_3/I_1 ratio starts to increase obviously[5]. The two vertical dashed lines indicate the concentration of 4.0 mM. It seems that the four systems have similar aggregation behavior with the CAC estimated to be ~4.0 mM for $CdCl_2$.The aggregation behavior should be dominated by the Cd–MPA complex (a, blue circular symbols).

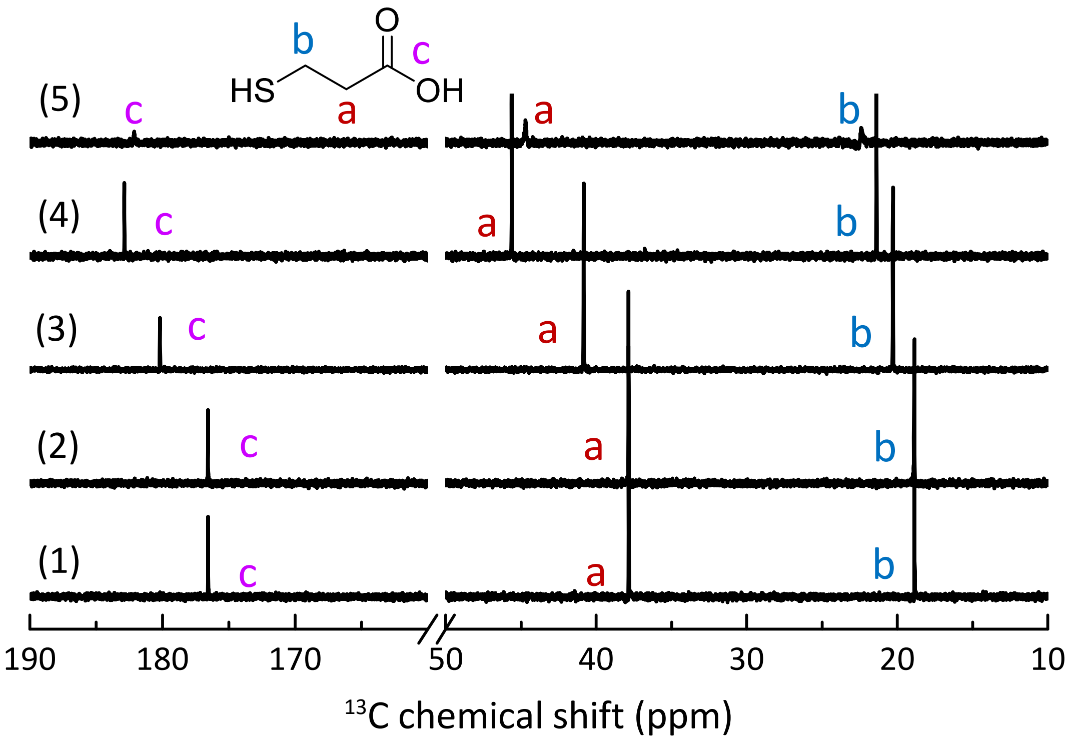

Supplementary Figure 15. The corresponding ^{13}C NMR spectra of the five solutions shown in Fig. 4. The solutions consist of 8MPA (1), 2$CdCl_2$-8MPA (2), 8MPA-8KOH (3), 8MPA-20KOH (4), and 2$CdCl_2$-8MPA-20KOH (5). The spectra are collected at room temperature. The labels *a*, *b* and *c* represent the carbon atoms in the MPA molecule as indicated. From Solutions 1 to 3 to 4 with the increase of the KOH concentration, the ^{13}C NMR of MPA shift downfield gradually. From Solutions 1 to 2 upon the presence of $CdCl_2$, the ^{13}C NMR of MPA changes little, suggesting that $CdCl_2$ and MPA have little coordination interaction in an acidic environment (pH ~3.0). From Solutions 4 to 5, however, the ^{13}C NMR signals broaden significantly (with Carbon b down-field shifting), suggesting also the aggregation of the Cd–MPA complex.

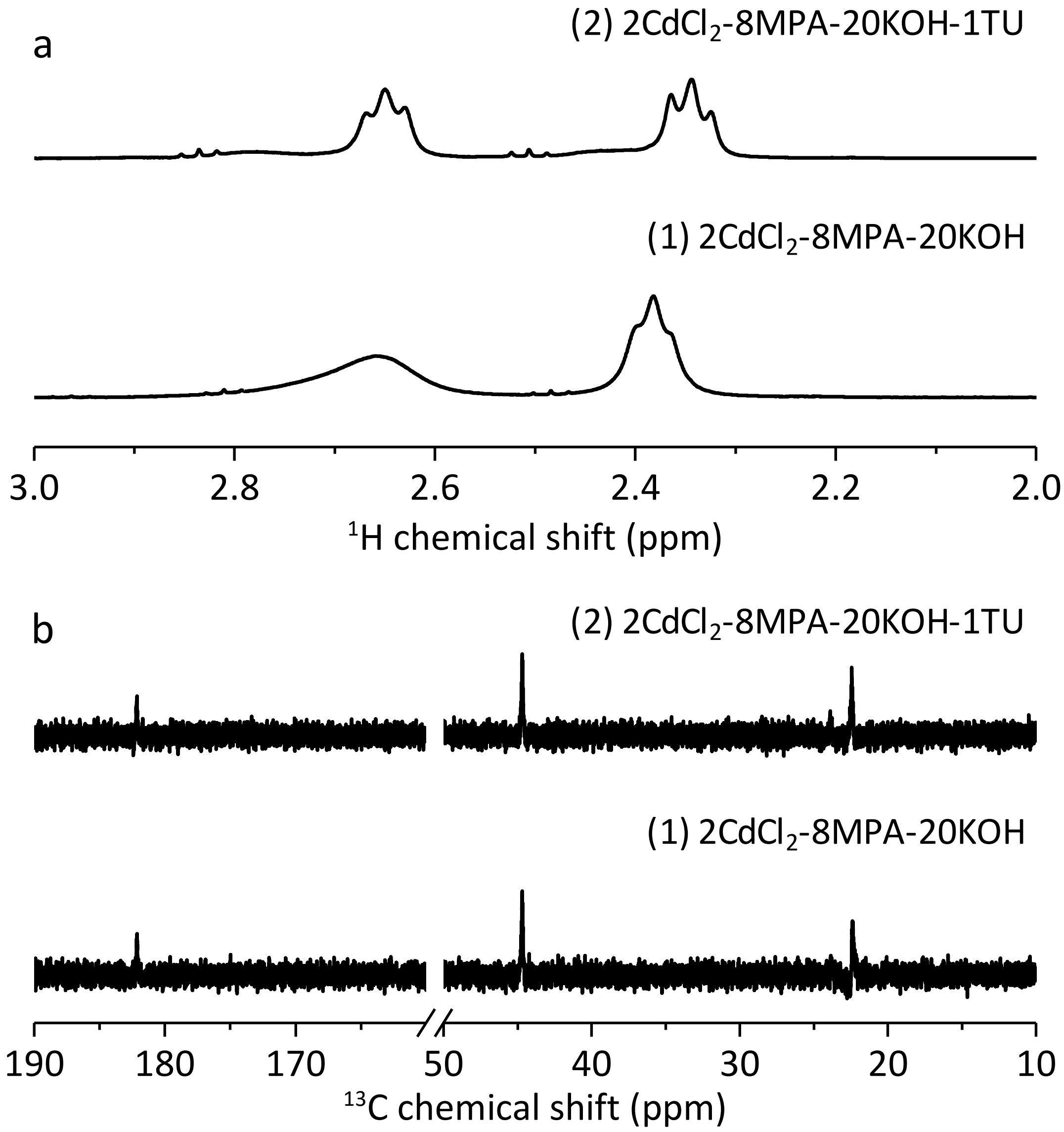

Supplementary Figure 16. ^{1}H (a) and ^{13}C NMR (b) spectra of two mixtures with feed molar ratios of 2CdCl$_2$-8MPA-20KOH (1) and 2CdCl$_2$-8MPA-20KOH-1TU (2). The MPA concentration is 40.0 mM in D_2O. The ^{1}H and ^{13}C NMR spectra are collected at room temperature. The ^{1}H and ^{13}C resonance signals of MPA change little upon the presence of TU.

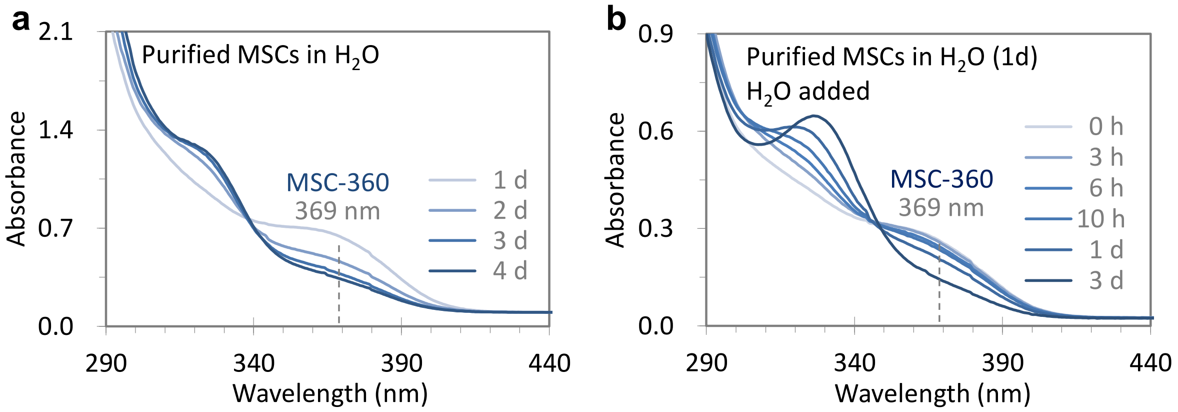

Supplementary Figure 17. Absorption spectra collected for the Fig. 5a dispersion over a period of 4 days (a), and after the 1-day dispersion (1.5 mL) is diluted with deionized water (1.5 mL) up to 3 days (b). The spectra are offset to have similar absorbance at 440 nm. The dashed lines signify the 369 nm position. The absorbance at 369 nm decreases for the two dispersions.

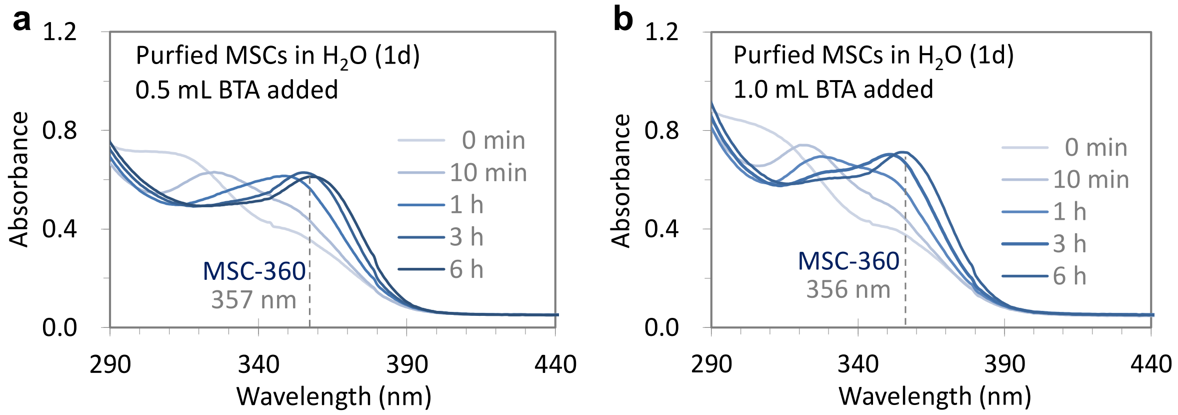

Supplementary Figure 18. Absorption spectra collected from the Fig. 5a 1-day dispersion, 1.5 mL of which is diluted with 1.0 mL water first and then 0.5 mL BTA (a), and with 0.5 mL water first and then 1.0 mL BTA (b). The spectra are offset to have similar absorbance at 440 nm. The dashed lines signify the positions indicated. The recovery of CdS MSC-360 is detected, similar to that shown in Fig. 5b.

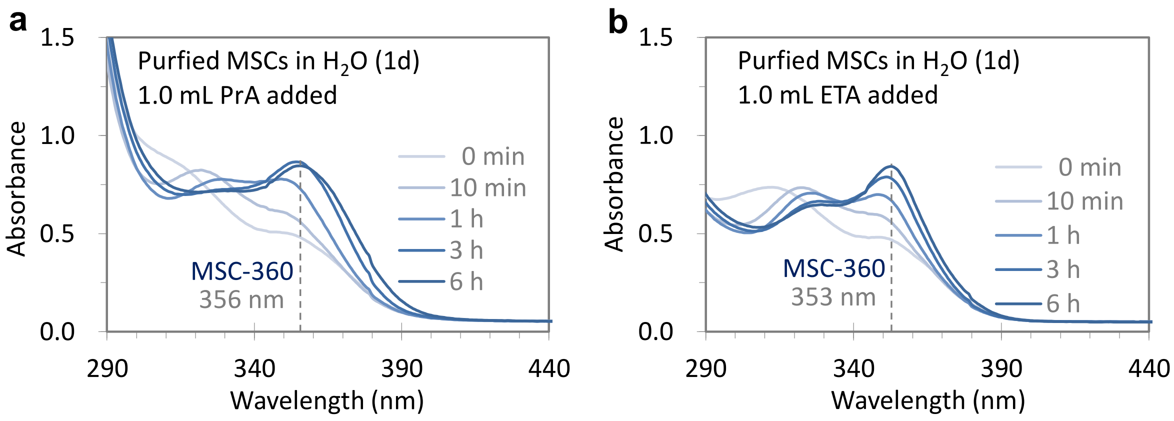

Supplementary Figure 19. Absorption spectra collected from the Fig. 5a 1-day dispersion, 1.5 mL of which is diluted with 0.5 mL water first and then 1.0 mL PrA (a), and with 0.07 mL water first and then 1.43 mL ETA (b). Note that 1.43 mL ETA aqueous solution (70%) has the effective volume of ETA about 1.0 mL. The spectra are offset to have similar absorbance at 440 nm. The dashed lines signify the positions pointed out. Evidently, both the primary amines, PrA and ETA, are able to induce the PC to MSC-360 transformations as well.

a

b

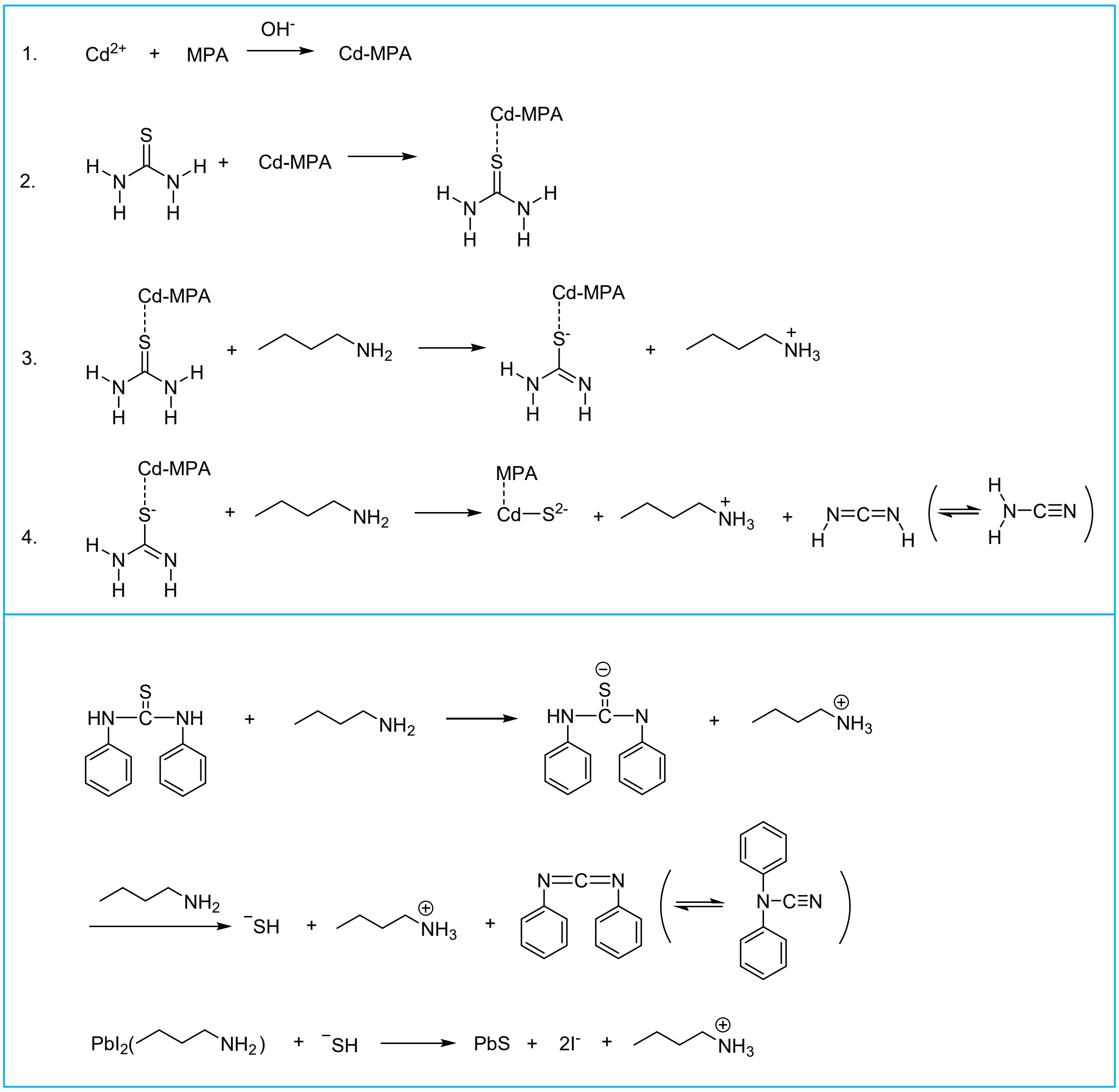

Supplementary Figure 20. (a) The reaction pathway proposed for the BTA-assisted decomposition of TU that results in the formation of Cd-S covalent bonds in the present room-temperature aqueous-phase approach to CdS MSCs. Step 1 addresses the formation of Cd–MPA complexes[6,7]. Step 2 deals with the coordination of TU and the resulting Cd–MPA complex. It is noteworthy that Supplementary Fig. 10 indicates clearly that without $CdCl_2$, TU remains stable in the aqueous solution with BTA and MPA. Hence, the BTA-assisted TU decomposition comes from BTA acting in concert with the Cd–MPA complex. Steps 3 and 4 follow those proposed for the BTA-assisted decomposition of diphenyl thiourea in dimethyl formamide (DMF)[8]. (b) Supplementary Figure 1 of Ref 8 (Ref 43 in our main text) is reproduced with ChemDraw to achieve an improved resolution, which shows the reaction between diphenyl thiourea (DPhTA), butylamine (BA), and lead iodide (PbI_2). This study suggests that without the involvement of the Pb precursor, the direct decomposition of

diphenyl thiourea resulted in HS^- as the S precursor, which then reacted with the Pb precursor PbI_2.

Supplementary Table 1. Summary of literature reports on the aqueous-phase syntheses of CdSe MSCs.

Refs	Cd sources	Se sources	Absorption peak (nm)
9-12	$CdSO_4$<br>L-cysteine	Na_2SeSO_3	420, 390, 360
13	$CdCl_2$<br>L-cysteine	NaHSe in ethanol	420, 390, 360
14-16	$Cd(OH)_2$<br>L-cysteine<br>OH^-	Na_2SeSO_3	422, 390, 360

Supplementary Table 2. Summary of literature reports on the aqueous-phase syntheses of CdE (E = S, Se and Te) QDs.

Refs	Cd sources	E sources (E = S, Se and Te)	Absorption peak (nm)
17	$Cd(ClO_4)_2 \cdot 6H_2O$<br>1-thioglycerol	H_2S	280-333
18	$CdCl_2$<br>TGA	Na_2S	~320-430
19	$CdCl_2 \cdot 2.5H_2O$<br>MPA	thiourea	363-409
20	$Cd(ClO_4)_2 \cdot 6H_2O$<br>R-SH	NaHSe	~450-530
21	$Cd(ClO_4)_2$<br>Sodium citrate	N,N-dimethylselenourea	~520-550
22	$CdCl_2 \cdot 2.5H_2O$<br>PVA	Na_2SeSO_3	~480-520
23	$Cd(ClO_4)_2 \cdot 6H_2O$<br>R-SH	H_2Te	~430-600
24	$CdCl_2$<br>MPA	NaHTe	~425-525
25	$CdCl_2$<br>MPA	NaHTe	480-615
26	$CdCl_2$<br>MPA	Na_2TeO_3	~500-580

TGA: thioglycolic acid; MPA: 3-mercaptoproponic acid; PVA: poly(vinyl alcohol).

Supplementary Note 1 Additional explanation for Supplementary Figure 4.

The TEM images (a and b) suggest that the as-synthesized and purified CdS MSC-360 has a spherical shape with a diameter less than 3 nm. It is well known that conventional characterization tools, such as TEM, cannot be used to reveal the actual crystalline structure in very small particles like colloidal semiconductor small-size QDs and MSCs[27,28].

The five powder XRD patterns (c) contain those for the purified CdS MPA capped MSC-360 (blue trace, with a scan rate of 1° per min), oleylamine (OLA) capped MSC-361 (via an organic-phase approach, gray trace from literature)[27], phenyl acetate (PA) capped MSC-322 (via an organic-phase approach, light gray trace from literature)[28], bulk zinc blende/cubic CdS (black vertical lines at the bottom), and bulk wurtzite/hexagonal CdS (black vertical lines at the top) [28]. Here, XRD demonstrates that aqueous-phase MSC-360 and organic-phase MSC-361 have somewhat similar structures, which appear to be different from that of MSC-322. These CdS MSCs have broad XRD peaks due to their limited number of atoms; when the QDs are small enough, no meaningful identification of their crystalline structures can be obtained with this method[29].

TGA results (d) (normalized at 250 °C) of purified MPA-capped CdS MSC-360 (blue trace), and oleate (OA) capped CdS QD-358 (gray trace) and OA-capped CdS MSC-322 (light gray trace). The latter two traces are from published literature, for which the QDs and MSC-322 had been synthesized via organic-phase approaches[28]. Each curve in the temperature range from 250 to 500 °C is a part of a TGA measurement from room temperature (for the former) or 30 °C (for the latter two) to 800 °C, with a heating rate of 5 °C per min for the former and of 15 °C per min for the latter two. All of the TGA results were obtained with the samples in a N_2 atmosphere. The weight loss in the temperature range of 250 to 450 °C for OA-capped MSC-322 was attributed to the decomposition of the surface capping ligands, and a weight ratio of 45 for the OA ligand to 55 for the inorganic core was estimated[28]. It seems that the OA-capped QD-358 and OA-capped MSC-322 both undergo a two-step process in the thermal decomposition of the surface OA ligand. We have placed two vertical dashed lines in Part c at 320 and 400 °C, as a guide to the eye and to highlight the two bumps that occur for the purified MPA-capped CdS MSC-360 sample. Similarly, we argue that the weight loss in the temperature range from 250 to 500 °C, which gives rise to the

two bumps, is due only to a similar process of thermal decomposition of the surface MPA ligand. TGA (in Part d) indicates that for the MPA-capped MSC-360 sample, the weight ratio of MPA ligand to inorganic core is about 20 to 80. Since the molecular weight of MPA (M_w = 106.1) is less than half of that of oleic acid (M_w = 282.5), it is reasonable that the ligand to inorganic core weight ratio for MSC-360 would be smaller than that for MSC-322.

Supplementary References

1. Liang, Y., Yu, K., Wang, J., Chen, J., Sun, B., Shao, L. Erythorbic acid promoted synthesis of CdS quantum dots in aqueous solution and study on optical properties. *Colloids Surf., A* **455**, 129–135 (2014).
2. Al-Abadleh, H. A., Grassian, V. H. FT-IR study of water adsorption on aluminum oxide surfaces. *Langmuir* **19**, 341–347 (2003).
3. Yang, J., Xue, C., Yu, S. H., Zeng, J. H., Qian, Y. T. General synthesis of semiconductor chalcogenide nanorods by using the monodentate ligand n-butylamine as a shape controller. *Angew. Chem. Int. Ed.* **41**, 4697–4700 (2002).
4. Zhu, T*., et al.* Two-step nucleation of CdS magic-size nanocluster MSC–311. *Chem. Mater.* **29**, 5727–5735 (2017).
5. Zhang, M., Fives, C., Waldron, K. C., Zhu, X. X. Self-assembly of a bile acid dimer in aqueous solutions: From nanofibers to nematic hydrogels. *Langmuir* **33**, 1084–1089 (2017).
6. Zhou, D., et al. Hydrazine-mediated construction of nanocrystal self-assembly materials. *ACS Nano* **8**, 10569–10581 (2014).
7. Han, J., et al. Growth kinetics of aqueous CdTe nanocrystals in the presence of simple amines. *J. Phys. Chem. C* **114**, 6418–6425 (2010).
8. Wang, Y*., et al.* Room-temperature direct synthesis of semi-conductive PbS nanocrystal inks for optoelectronic applications. *Nat. Commun.* **10**, 5136 (2019).
9. Park, Y.-S*., et al.* Aqueous phase synthesized CdSe nanoparticles with well-defined numbers of constituent atoms. *J. Phys. Chem. C* **114**, 18834–18840 (2010).
10. Park, Y.-S*., et al.* Size-selective growth and stabilization of small CdSe nanoparticles in aqueous solution. *ACS Nano* **4**, 121–128 (2010).
11. Baker, J. S., Nevins, J. S., Coughlin, K. M., Colón, L. A., Watson, D. F. Influence of complex-formation equilibria on the temporal persistence of cysteinate-functionalized CdSe nanocrystals in water. *Chem. Mater.* **23**, 3546–3555 (2011).
12. Park, Y.-S., Okamoto, Y., Kaji, N., Tokeshi, M., Baba, Y. Aqueous phase synthesized CdSe magic-sized clusters: Solution composition dependence of adsorption layer structure. *J. Nanosci. Nanotechnol.* **12**, 539–546 (2012).
13. Deng, D., Yu, J. Spontaneous transformation of CdSe nanoparticles into nonspherical Se crystals: Role of the precursor ligand. *Cryst. Growth Des.* **15**, 602–609 (2015).
14. Kurihara, T., Noda, Y., Takegoshi, K. Quantitative solid-state NMR study on ligand-surface interaction in cysteine-capped CdSe magic-sized clusters. *J. Phys. Chem. Lett.* **8**, 2555–2559 (2017).
15. Kurihara, T., Matano, A., Noda, Y., Takegoshi, K. Rotational motion of ligand-cysteine on CdSe magic-sized clusters. *J. Phys. Chem. C* **123**, 14993–14998 (2019).
16. Kurihara, T., Noda, Y., Takegoshi, K. Capping structure of ligand–cysteine on CdSe magic-sized clusters. *ACS Omega* **4**, 3476–3483 (2019).
17. Vossmeyer, T*., et al.* CdS Nanoclusters: synthesis, characterization, size dependent oscillator strength, temperature shift of the excitonic transition energy, and reversible absorbance shift. *J. Phys. Chem.* **98**, 7665–7673 (1994).
18. Zheng, J*., et al.* Correlation between the photoluminescence and oriented attachment growth mechanism of CdS quantum dots. *J. Am. Chem. Soc.* **132**, 9528–9530 (2010).
19. Aboulaich, A*., et al.* One-pot noninjection route to CdS quantum dots via hydrothermal synthesis. *ACS Appl. Mater. Interfaces* **4**, 2561–2569 (2012).

20. Rogach, A. L., Kornowski, A., Gao M., Eychmüller, A., Weller, H. Synthesis and characterization of a size series of extremely small thiol-stabilized CdSe nanocrystals. *J. Phys. Chem. B* **103**, 3065–3069 (1999).
21. Rogach, A. L., Nagesha, D., Ostrander, J. W., Giersig, M., Kotov, N. A. "Raisin bun"-type composite spheres of silica and semiconductor nanocrystals. *Chem. Mater.* **12**, 2676–2685 (2000).
22. Ma, X. D., Qian, X. F., Yin, J., Xi, H. A., Zhu, Z. K. Preparation and characterization of polyvinyl alcohol-capped CdSe nanoparticles at room temperature. *J. Colloid Interface Sci.* **252**, 77–81 (2002).
23. Gaponik, N.*, et al.* Thiol-capping of CdTe nanocrystals: An alternative to organometallic synthetic routes. *J. Phys. Chem. B* **106**, 7177–7185 (2002).
24. Zhang, H., Wang, D., Yang, B., Mohwald, H. Manipulation of aqueous growth of CdTe nanocrystals to fabricate colloidally stable one-dimensional nanostructures. *J. Am. Chem. Soc.* **128**, 10171–10180 (2006).
25. Han, J., Luo, X., Zhou, D., Sun, H., Zhang, H., Yang, B. Growth kinetics of aqueous CdTe nanocrystals in the presence of simple amines. *J. Phys. Chem. C* **114**, 6418–6425 (2010).
26. Zhou, D.*, et al.* Simple synthesis of highly luminescent water-soluble CdTe quantum dots with controllable surface functionality. *Chem. Mater.* **23**, 4857–4862 (2011).
27. Tang, J.*, et al.* CdS magic-size clusters exhibiting one sharp ultraviolet absorption singlet peaking at 361 nm. *Nano Res.* **12**, 1437–1444 (2019).
28. Zhang, B.*, et al.* Thermally-induced reversible structural isomerization in colloidal semiconductor CdS magic-size clusters. *Nat. Commun.* **9**, 2499 (2018).
29. Murray, C. B., Norris, D. J., Bawendi, M. G. Synthesis and characterization of nearly monodisperse CdE (E = S, Se, Te) semiconductor nanocrystallites. *J. Am. Chem. Soc.* **115**, 8706–8715 (1993).
